# Anti-PLA2R autoantibodies induce complement activation via IgG subclass–dependent epitope pairings in membranous nephropathy

**DOI:** 10.1172/JCI191995

**Published:** 2026-09-08

**Authors:** Tsai-Yi Wu, Kun-Hua Tu, Larissa Seifert, Kung-Wei Lin, Han-Po Shih, Yu-Fang Lo, Jhan-Jie Peng, Gunther Zahner, Oliver Kretz, You-Ning Lin, Chen-Xuan Kang, Jing-Ya Ding, Yi-Ran Tu, Li-Yi Ma, Ya-Ting Chuang, Chia-Chi Lo, Yu-Huan Tsai, Chih-Wei Yang, Nicola M. Tomas, Cheng-Lung Ku

**Affiliations:** 1 Laboratory of Human Immunology and Infectious Disease, Institute of Immunology and Translational Medicine, College of Medicine, Chang Gung University, Taoyuan, Taiwan.; 2 III. Department of Medicine, and; 3 Hamburg Center for Kidney Health (HCKH), University Medical Center Hamburg-Eppendorf, Hamburg, Germany.; 4 Graduate Institute of Clinical Medical Sciences, College of Medicine, Chang Gung University, Taoyuan, Taiwan.; 5 Kidney Research Center, Department of Nephrology, Chanα-g Gung Memorial Hospital, Taoyuan, Taiwan.; 6 Institute of Microbiology and Immunology, School of Life Sciences, National Yang Ming Chiao Tung University, Taipei, Taiwan.; 7 Center for Molecular and Clinical Immunology, College of Medicine, Chang Gung University, Taoyuan, Taiwan.; 8 Division of General Surgery, Department of Surgery, Chang Gung Memorial Hospital, Taoyuan, Taiwan.; 9 Core Facility for Electron Microscopy, University Medical Center Hamburg-Eppendorf, Hamburg, Germany.; 10 Department of Medical Research, National Taiwan University Hospital, Taipei, Taiwan.; 11 Laboratory of Host-Microbe Interactions and Cell Dynamics, Department of Medical Biotechnology and Laboratory Science, Chang Gung University, Taoyuan, Taiwan.; 12 Institute of Immunology and Translational Medicine, College of Medicine, Chang Gung University, Taoyuan, Taiwan.; 13 Hamburg Center for Translational Immunology (HCTI), University Medical Center Hamburg-Eppendorf, Hamburg, Germany.; 14 Division of Infectious Diseases, Department of Pediatrics, Chang Gung Memorial Hospital, Taoyuan, Taiwan.; 15 Center for Drug Research and Development, College of Human Ecology, Chang Gung University of Science and Technology, Taoyuan, Taiwan.

**Keywords:** Autoimmunity, Immunology, Nephrology, Autoimmune diseases

## Abstract

Membranous nephropathy (MN) is an autoimmune kidney disease and a major cause of nephrotic syndrome in adults. Although autoantibodies against phospholipase A2 receptor 1 (PLA2R) and complement activation are central to disease pathogenesis, the mechanisms by which anti-PLA2R antibodies activate complement at the podocyte surface remain incompletely defined. Here, we cloned 14 patient-derived anti-PLA2R mAbs and found that they predominantly recognized the N-terminal cysteine-rich (CysR) and C-type lectin domain 1 (CTLD1) regions of PLA2R. Individual anti-PLA2R mAbs induced little or no complement-dependent cytotoxicity (CDC) of PLA2R-expressing podocytes in vitro. In contrast, paired mAbs targeting distinct epitopes, particularly CysR and CTLD1, markedly enhanced CDC. This effect was strongest for IgG1 and IgG3 antibodies, whereas IgG4 alone did not activate complement but modulated CDC in combination with IgG1. Purified IgG from patients with PLA2R-associated MN similarly induced CDC, which was augmented by addition of anti–PLA2R IgG1 and reduced by anti–PLA2R IgG4 or Fab fragments targeting CysR or CTLD1. In human PLA2R–expressing mice, paired anti-PLA2R antibodies increased glomerular complement deposition and induced albuminuria. These findings identify epitope pairing as a key determinant of complement activation in PLA2R-associated MN and support epitope-specific targeting strategies as a promising avenue for therapeutic intervention.

## Abstract 2

This study demonstrates that anti-PLA2R autoantibodies activate complement in pMN via the synergy of IgG1/IgG3 epitope pairings, with specific epitopes of IgG4 reducing this effect.<br> <br>

## Introduction

Membranous nephropathy (MN) is an organ-specific, immune-mediated glomerular disorder characterized by the subepithelial deposition of immune complexes and complement factors (1). Approximately 20% of MN cases are associated with conditions such as systemic lupus erythematosus (SLE), hepatitis B virus, malignancies, or exposure to infections and drugs and are therefore classified as secondary MN (2). Up to 80% of MN is primary MN, which is predominantly defined by autoantibodies targeting proteins in the podocyte plasma membrane, with the phospholipase A2 receptor (PLA2R) antigen being linked to 75% of cases (3). Other identified antigens in MN include thrombospondin type 1 domain–containing protein 7A (THSD7A) (4), neural EGF-like 1 (NELL-1) (5), semaphorin 3B (SEMA3B) (6), protocadherin 7 (PCDH7) (7), high-temperature requirement A serine peptidase 1 (HTRA1) and others (8).

The PLA2R protein consists of an N-terminal cysteine-rich domain (CysR), followed by a fibronectin type II domain (FNII), and 8 C-type lectin–like domains (CTLD1–CTLD8) (9). Its presence has been confirmed in podocytes, neutrophils (10), and respiratory epithelial cells (11). PLA2R binds to secretory phospholipase A2 group IB in circulation with high affinity and acts as a signaling molecule to promote human podocyte injury (12, 13). However, its exact function in the kidneys remains unclear. In addition to autoantibodies targeting PLA2R, patients with MN show increased PLA2R protein in the glomerulus with no significant difference in mRNA levels (14). Transgenic mouse models with podocyte-specific overexpression of PLA2R showed that PLA2R expression and the presence of anti-PLA2R autoantibodies can result in proteinuria (15–17). Anti-PLA2R autoantibodies primarily bind to CysR and CTLD1, but autoantibodies binding CTLD7 and CTLD8 are also frequently present (18–20). Anti-PLA2R autoantibodies in patients with MN are predominantly IgG4 (3), the IgG subclass with the least C1q-binding capacity and thus limited (or no) ability to activate the complement system via the classical pathway (21). However, IgG1 and IgG3, the IgG subclasses with the highest capacity to bind C1q and activate complement via the classical pathway, are also present in both the serum and glomerular immune deposits of patients with MN (22–25). In line with this, glomerular C1q is also regularly positive in immunohistology (25, 26). Interestingly, contemporary research posits that anti–PLA2R IgG4, via MBL binding, activates the lectin pathway, inducing alterations in podocyte cytoskeletal dynamics (27, 28).

Taken together, IgG subclass distribution and complement deposition are well described in MN, but how autoantibodies against different PLA2R epitopes synergize to activate the complement system is incompletely understood. Here, we investigated the molecular basis of complement activation in MN using patient-derived anti-PLA2R1 mAbs and demonstrate that anti-CysR and anti-CTLD1 antibodies synergistically acted to activate the complement system both in vitro and in vivo.

## Results

### Isolation of anti-PLA2R mAbs from the blood of patients with PLA2R-associated MN.

To elucidate the complement activation capacities of human anti-PLA2R autoantibodies, we isolated 14 anti-PLA2R mAbs using flow cytometry–based single B cell sorting of blood from 4 patients with MN (Table 1). All patients tested positive for anti-PLA2R antibodies according to a commercial ELISA kit (Figure 1A). According to the 2021 Kidney Disease Improving Global Outcomes (KDIGO) clinical practice guidelines (29), 17 of 73 (23%) patients with MN had antibody concentrations within the non-high-titer range and 16 of 73 (22%) patients exceeded the high-titer threshold. Four patients with MN who had a high titer of anti-PLA2R antibodies were selected for antibody cloning. In these patients, IgG4 was found to be the dominant circulating anti-PLA2R IgG subclass, but IgG1 and IgG3 were also present (Figure 1B).

We then used recombinant soluble PLA2R (Supplemental Figure 1; supplemental material available online with this article; https://doi.org/10.1172/JCI191995DS1) as bait to isolate single, PLA2R-specific B cells via a single-cell sorting platform (Supplemental Figure 2) (30). We isolated 120 memory B cells and their B cell receptor repertoire was subcloned into IgG1 plasmids and expressed in HEK cells. Fourteen of the mAb clones showed binding to PLA2R by both ELISA and Western blotting (Figure 1, C and D).

Genetic analysis of the 14 mAbs provided their IgH, IgK, and IgL sequences. Of the 14 mAbs, 6 were IgG4, 4 were IgG1, 2 were IgG3, and 2 were IgG2, and κ was the predominant light chain. The mAbs 2G7, 2F10, and 2H2, sharing the IGHV1-69-D2-15-J6 and IGKV3-20-J4 coding sequences, were identified as identical clonotypes originating from a single donor, albeit ones that exhibited variations in somatic hypermutation. Furthermore, the J1-1 region within the IGKJ gene emerged as the predominant group in 10 of the 14 mAb clones, but no dominant V region gene was found (Table 1). The 14 mAbs had undergone somatic mutations, with an average of 14.4 ± 2.3, 9.7 ± 3.2, and 13.5 ± 1.5 mutations in the IgH, Igκ, and Igλ V genes, respectively. Compared with healthy controls, the average mutation rates in the IgH and Igκ V genes were similar, while the mutation rate in the Igλ V genes was twice as high, suggesting the involvement of T cell–dependent B cell maturation (31). Together, these experiments show the successful generation of patient-derived anti-PLA2R mAbs.

### Epitope mapping of patient-derived mAbs.

We mapped the epitope regions bound by the mAbs using a series of truncated PLA2R versions (ΔF–Δ7 and the CysR singular domain; Figure 2A). Western blot assays revealed that the 14 mAbs recognized 3 distinct domains: CysR (7 clones), CTLD1 (6 clones), and CTLD6 (1 clone) (Figure 2, A and B, and Supplemental Figure 3). The high prevalence of CysR- and CTLD1-reactive mAbs is consistent with studies analyzing serum from patients with MN, in which CysR and CTLD1 were identified as major binding domains (18, 32). We further performed a cross-competition assay with sandwich orientation through biolayer interferometry (BLI) to analyze potential competition for PLA2R binding. These epitope binning experiments demonstrated that several of the mAbs compete for binding to CysR and CTLD1 (Figure 2C). Of note, the CysR-reactive anti-PLA2R mAbs 2G7 and 1G5, as well as the CTLD1-reactive anti-PLA2R mAb 1E6 exhibited binding interference between the CysR and CTLD1 domains (Figure 2C). The CysR-reactive mAb 2G7, as a representative example, showed binding interference with most mAbs targeting CTLD1 (4 of 6), suggesting that the binding site of 2G7 spans from the CysR to the CTLD1 domain, likely due to the spatial proximity of these 2 domains within PLA2R. In contrast to the 2G7, 1G5, and 1E6 mAbs, the other 11 mAbs exhibited restricted binding epitopes and only occasionally interfered with the binding of mAbs targeting the same domain (Figure 2C and Supplemental Figure 4).

The affinities of these mAbs for PLA2R were characterized by equilibrium dissociation constants (*K_D_*) ranging from 1.16 × 10^–8^ to 3 × 10^–11^ M, as measured by BLI. The presence of somatic hypermutations and the binding affinities of these antibodies, ranging from 10^–8^ to 10^–11^ M, were consistent with established benchmarks for autoantibodies (30). The observed kinetic association rates (*k_a_*) ranged from 8.3 × 10^4^ to 8.6 × 10^5^ 1/Ms, while the binding affinities, quantified by kinetic dissociation rates (*k_d_*), were between 7.18 × 10^–6^ and 9.93 × 10^–3^ 1/s (Table 2 and Supplemental Figure 5). Of note, the *K_D_* metrics for the IgG1 Fc clones were mostly lower than 10^–9^ M, implying superior affinity compared with the IgG4 Fc clones, which were all higher than 10^–9^ M (Figure 2D).

In patients with primary MN, IgG4 antibodies colocalize with PLA2R at the glomerular filtration barrier (3). We therefore assessed the binding of the anti-PLA2R mAbs to membrane-expressed PLA2R by staining of PLA2R-EGFP–overexpressing HEK293 cells. Anti-PLA2R mAb staining colocalized with PLA2R-EGFP signals, demonstrating that the anti-PLA2R mAbs also recognized PLA2R when expressed on the cell surface (Supplemental Figure 6A).

Additionally, we established a podocyte cell line (AB8/13) that stably overexpressed membrane PLA2R-EGFP through lentivirus-mediated transduction followed by FACS (further referred to as PLA2R-AB8/13 or PLA2R-expressing podocytes). PLA2R was expressed in both differentiated and undifferentiated podocytes (Supplemental Figure 6B), and anti-PLA2R mAbs, but not control IgG, bound to PLA2R-expressing podocytes (Supplemental Figure 6C). Together, these results demonstrate that anti-PLA2R mAbs derived from patients with MN, although exhibiting diverse binding affinity, can bind to both soluble PLA2R and membrane-expressed PLA2R and favor the CysR and CTLD1 regions, irrespective of the subclass.

### Complement-dependent target cell lysis of individual mAbs.

As complement activation is a central pathogenic driver of MN (33), we evaluated the capacity of different anti-PLA2R IgG subclasses to initiate complement activation by complement-dependent cytotoxicity (CDC) assays. We found that all anti-PLA2R mAbs of the IgG4 subclass had no effect on cell viability of both WT and PLA2R-expressing podocytes (Figure 3A). We then reengineered the anti-PLA2R mAbs as IgG1, which can bind C1q and activate the classical complement pathway. Interestingly, we observed that the 1E12 mAb, which recognizes CTLD1, induced moderate CDC of PLA2R-expressing podocytes, resulting in 76% cell viability (Figure 3A). This effect was specific for the IgG1 Fc backbone, as the IgG4 variant showed no CDC effect (Figure 3B).

To further investigate CDC by anti-PLA2R mAbs, we selected the representative clones 2B8 and 1B7 (both CysR reactive) as well as 1B10 and 1E12 (both CTLD1 reactive) and found that 1E12 induced substantial target cell lysis at a concentration of 50 ng/mL or higher. Additionally, 2B8, but not 1B10 or 1B7, induced moderate CDC activation, leading to 17% cell lysis at higher concentrations (Figure 3C). CDC induced by the 1E12 and 2B8 mAbs was abrogated by heat inactivation of the serum that was used as a source of complement (Figure 3C). These findings indicate that most anti-PLA2R mAbs, even if in an IgG1 format, typically do not induce higher-degree, complement-dependent podocyte lysis.

### Anti-PLA2R mAb epitope pairing induces CDC.

We next investigated the potential synergistic effects of antibodies targeting distinct epitopes in CysR and CTLD1, the major epitope-containing domains in PLA2R (20, 32, 34). Although 1B10, reactive with CTLD1, did not induce CDC of mPLA2R-podocytes alone (Figure 3C), the addition of 2B8, reactive with CysR, resulted in significant lysis of 59% of target cells (Figure 3D). On the basis of this observation and the initial epitope binning results (Figure 2C), we selected mAbs with confirmed cobinding capacity for a detailed systematic functional assessment.

The combination of 2 anti-PLA2R mAbs targeting the same domain, either CysR (2B8 and 1B7) or CTLD1 (1B10 and 2F1), resulted in increased CDC compared with the effect of each mAb alone, with 38% and 36% of target cells lysed, respectively (Figure 3D). The combinations of 2 mAbs targeting 2 different domains, specifically 2B8 (CysR reactive) and 1B10 (CTLD1 reactive), elicited a significantly higher level of CDC than did combinations targeting the same domain, and we observed this enhancement in a dose-dependent manner (Figure 3D).

We further investigated whether combining 3 mAbs that can independently bind to PLA2R could further enhance CDC. The combinations included (a) 1 CysR-reactive mAb (2B8) with 2 CTLD1-reactive mAbs (1B10 and 2F1), (b) 1 CTLD1-reactive mAb (1B10) with 2 CysR-reactive mAbs (2B8 and 1B7), and (c) 1 CTLD6-reactive mAb (2B12) with both a CTLD1-reactive (1B10) mAb and a CysR-reactive mAb (2B8). None of these triple combinations induced a CDC that was higher than the 2B8-1B10 pair (Figure 3D). Notably, the enhanced CDC observed with the CTLD1-reactive mAb 1B10 was consistently reproduced when combined with any CysR-reactive mAb or with the CTLD1-reactive mAb 2F1, but not when combined with CTLD1-reactive mAbs that lacked cobinding ability (Figure 3E). Similarly, 2B8 enhanced CDC when paired with any CTLD1-reactive mAb or with the CysR-reactive mAb 1B7, but not when combined with CysR-reactive mAbs that lacked cobinding ability (Figure 3F). Importantly, we observed no target cell lysis after heat inactivation of the serum that was used as the source of complement (Supplemental Figure 7). Together, these results demonstrate that, in vitro, pairing of anti-PLA2R mAbs targeting distinct PLA2R domains synergistically enhanced CDC activity and that combining 3 mAbs with cobinding capacity did not further increase CDC when compared with combining only 2 mAbs.

### Epitope pairing enhances complement activation and glomerular injury in vivo.

Human PLA2R–transgenic mouse models recapitulate key features of MN, including glomerular IgG and complement deposition (15–17). We used human PLA2R–expressing recombination activating gene 2–deficient *(Rag2−/−)* mice to investigate whether epitope pairing determines complement activation and podocyte injury in vivo (16). We initially injected mice with either a single anti-PLA2R mAb (2B8 or 1B7 targeting CysR as well as 1B10 or 2F1 targeting CTLD1), a combination of 2 mAbs (2B8/1B7, 2B8/1B10, 1B10/2F1), a combination of 3 mAbs (2B8/1B7/1B10, 2B8/1B10/2F1), and a combination of 4 mAbs (2B8/1B7/1B10/2F1) (Figure 4A). All mAbs were on a mouse IgG2a (mIgG2a) backbone, which resembles human IgG1 in its capacity to bind C1q and activate the classical pathway of complement (35).

After injection, circulating levels of anti-PLA2R antibody were similar between the different groups, and the mice did not develop significant albuminuria during the 10-day observation period (Figure 4, B and C). We analyzed histology 10 days after mAb transfer for mIgG and C3 deposition. Confocal microscopy revealed a granular staining pattern for mIgG at the subepithelial aspect of the glomerular basement membrane, which increased with the number of injected mAbs (Figure 4, D and E, and Supplemental Figure 8). Notably, C3 was only scarcely detected in mice receiving 1 or 2 mAbs, was increasingly present in mice receiving 3 mAbs, and was markedly present in mice receiving 4 mAbs (Figure 4, F and G, and Supplemental Figure 8). Together, these experiments demonstrate that glomerular mIgG and complement deposition increased when mAbs targeting distinct epitopes in PLA2R were paired.

As MN is a disease of chronic glomerular immune complex accumulation (36), we immunized another cohort of mice 2 times with higher doses using the combination of 4 mAbs (Figure 5A), which had previously induced a most prominent glomerular complement deposition (Figure 4, F and G), and observed the mice for a total of 30 days. Serum samples collected on days 10 and 30 confirmed the presence of circulating anti-PLA2R antibodies (Figure 5B). Interestingly, mice injected with 4 anti-PLA2R mAbs developed albuminuria after the second injection, which increased over time and was significant toward the end of the observation period (Figure 5C). Confocal microscopy demonstrated significant deposition of mIgG and C3 along the glomerular capillary wall after 30 days (Figure 5, D–G). Electron microscopic analyses revealed podocyte foot process broadening and sparse electron-dense deposits at the subepithelial aspect of the glomerular basement membrane (GBM) in mice after receiving 4 mAbs, but not in control mice (Figure 5H). Together, these in vivo data provide evidence that epitope pairing and cobinding of multiple anti-PLA2R antibodies promoted complement activation and subsequent kidney injury.

### Epitope pairing by anti–PLA2R IgG1 and IgG3 mAbs potentiates complement activation.

Anti–PLA2R IgG1 and IgG3 are present in the vast majority of patients with PLA2R-associated MN in both the serum and in glomerular immune deposits (24, 25, 37), but how they activate the complement system is incompletely understood. IgG3, like IgG1, is known to bind C1q and induce complement activation via the classical pathway (24). We therefore tested the effect of IgG3 in CDC by converting the 2B8 and 1B10 mAbs into an IgG3 format. We found that combining 2 IgG3 mAbs, 2 IgG1 mAbs, or 1 IgG3 and 1 IgG1 mAb all induced similar levels of target cell lysis (Figure 6A).

As IgG4 is the dominant IgG subclass of anti-PLA2R autoantibodies (3), we investigated the effect of the IgG4 subclass on the synergistic activation of CDC when targeting paired epitopes (CysR and CTLD1). As expected, neither a single anti-PLA2R IgG4 mAb nor the combination of 2 anti-PLA2R IgG4 mAbs targeting distinct epitopes in PLA2R induced CDC (Figure 6B). However, both the CysR- and the CTLD1-directed IgG4 mAbs significantly enhanced CDC when paired with a CysR- or CTLD1-directed IgG1 mAb (Figure 6B and Supplemental Figure 9). This CDC activation by an IgG1-IgG4 pair was less prominent than that of 2 IgG1 molecules, but the experiments indicated that anti–PLA2R IgG4 could augment CDC when combined with an anti–PLA2R IgG1.

We then tested how the presence of anti–PLA2R IgG4 affects CDC induced by anti-IgG1 molecules using 2 different approaches. First, the combination of a CysR-targeted mAb (2B8) and a CTLD1-targeted mAb (1B10) was tested at different concentrations in CDC assays, in the presence of a control IgG4 or different anti-PLA2R IgG4 mAbs at fixed concentrations (10 μg/mL) (Figure 6C). Although the combination of the 2 anti–PLA2R IgG1 mAbs induced CDC at all the tested concentrations in the presence of a control IgG4, the addition of 1 or 2 anti-PLA2R IgG4 mAbs targeting the same epitope(s) significantly decreased target cell lysis (Figure 6C). This effect decreased with increasing concentrations of the 2 IgG1 mAbs. Second, the combination of a CysR- (2B8) and a CTLD1-targeted mAb (1B10) was tested at a fixed concentration (0.2 μg/mL), with IgG4 mAbs being added at increasing concentrations (Figure 6D). Although the combination of the 2 anti–PLA2R IgG1 mAbs induced stable CDC when a control IgG4 mAb was added, the addition of 1 or 2 anti–PLA2R IgG4 mAbs decreased CDC in a dose-dependent manner (Figure 6D). In summary, these experiments show that, although anti–PLA2R IgG4 may support IgG1 in activating the classical pathway of complement, its capacity to do so was less pronounced than that of IgG1.

### Anti-PLA2R–containing IgG from patients with MN induces CDC through epitope pairings by IgG1/3.

We further investigated the synergistic effect of plasma anti-PLA2R autoantibodies from patients with MN. To this end, we purified the IgG fraction from the plasma of 12 patients with PLA2R-associated MN and 10 control individuals. IgG4 was the most abundant anti-PLA2R subclass in the purified IgG fraction, but IgG1 and IgG3 were also present (Supplemental Figure 10A). While neither control nor patient IgG induced lysis of nontransduced target cells (AB8/13), the patients’ IgG induced significant lysis of PLA2R-expressing podocytes (PLA2R-AB8/13) in CDC assays, ranging from 12% to 52% (Figure 7A, left and middle panels). CDC was abolished if the serum that was used as the source of complement was heat inactivated (Figure 7A, right panel).

As discussed above, anti–PLA2R IgG1 and IgG3 were strong inducers of CDC when using mAbs in vitro (Figure 6A). To further explore this using patient plasma with a more natural composition of IgG subclasses, we next determined the CysR- and CTLD1-specific IgG subclasses. Direct ELISAs revealed high levels of IgG4 antibodies reactive to both CysR and CTLD1 (Figure 7B). Although IgG1 and IgG3 antibodies reactive to CysR were detected in all patients except patient 5, IgG1 and IgG3 antibodies reactive to CTLD1 were scarce in patients 5, 12, and 13 (Figure 7B), potentially explaining the lower CDC activity observed in these patients (Supplemental Figure 10B). To further study the synergistic role of IgG1 antibodies reactive with CysR and CTLD1, we added a CTLD1-reactive anti–PLA2R IgG1 mAb (1B10) to the CDC assay. The CTLD1-reactive anti–PLA2R IgG1 mAb, both alone and in combination with healthy donor IgG, did not induce significant target cell lysis (Figure 7C). However, the CTLD1-reactive anti–PLA2R IgG1 mAb in combination with a CysR-reactive anti-PLA2R mAb (2B8) and healthy donor IgG significantly enhanced CDC (Figure 7C). Moreover, the CTLD1-reactive mAb in combination with patient IgG (patients 5, 12, and 13) significantly enhanced CDC in a dose-dependent manner (Figure 7C), underscoring the role of CTLD1-reactive anti–PLA2R IgG1 autoantibodies in CDC activation, even in the absence of endogenous CTLD1-reactive anti–PLA2R-IgG1/IgG3 in MN.

To further investigate the synergistic effects of anti-CysR and anti-CTLD1 autoantibodies, we generated recombinant CysR and CTLD1 for neutralization assays. Both CysR and CTLD1 recombinant proteins significantly reduced CDC activity when preincubated with IgG purified from the plasma of patients with MN.

In light of the results that CysR- and CTLD1-reactive anti–PLA2R IgG4 mAbs reduced CDC by CysR- and CTLD1-reactive anti–PLA2R IgG1 mAbs (Figure 6, C and D), we next examined their effects on CDC mediated by patient IgG. Both the CysR-reactive IgG4 alone and the combination of the CysR- and the CTLD1-reactive mAbs significantly decreased CDC mediated by patient IgG (Figure 7E). Together, these findings highlight the critical role of CysR- and CTLD1-reactive anti–PLA2R IgG1/3 antibodies in triggering complement activation despite not being the predominant subclasses in the patients’ plasma.

### Fab fragment competition to reduce complement activation.

Achieving immunologic remission, defined as the disappearance of anti-PLA2R autoantibodies following immunosuppressive treatment, is an important therapeutic goal for patients with PLA2R-associated MN (38). However, current treatment strategies are not always associated with satisfactory outcomes. Drawing from our understanding that the synergistic effect of autoantibodies reactive to CysR and CTLD1 is essential for disease progression, we hypothesized that Fab fragments competitively binding to CysR or CTLD1 may attenuate CDC by anti-PLA2R autoantibodies. We generated Fab fragments from CysR-reactive (2B8) and CTLD1-reactive (1B10) mAbs and evaluated their effects on CDC activation by anti–PLA2R IgG1/3 from patients with MN whose antibodies were reactive to both CysR and CTLD1 (*n* = 8; Figure 8). The results demonstrated that both CysR- and CTLD1-directed Fab fragments significantly reduced target cell lysis when probed on 2 PLA2R-expressing podocyte lines (AB8/13 and LY; Figure 8, A and B). These findings highlight the critical role of synergy between CysR- and CTLD1-reactive anti-PLA2R autoantibodies as well as the importance of their Fc regions in driving complement activation in PLA2R-associated MN. Furthermore, they suggest that preventing PLA2R-autoantibody interactions on podocytes through competitive inhibition by Fab fragments may represent a potential therapeutic strategy to mitigate complement activation in patients with MN.

## Discussion

Autoantibodies targeting PLA2R are the central drivers of MN (39), yet the mechanisms by which these antibodies induce complement-mediated podocyte injury remain incompletely understood. In this study, we generated a panel of 14 patient-derived anti-PLA2R mAbs and used them to dissect how epitope specificity and IgG subclasses cooperate to drive complement activation. Our findings identified epitope pairing across the CysR and CTLD1 domains as a critical determinant of complement activation and highlight what we believe to be an underappreciated role of IgG1 and IgG3 autoantibodies in amplifying disease-relevant effector mechanisms.

Using single B cell cloning, we isolated mAbs spanning not only IgG4 but all 4 IgG subclasses that predominantly targeted the CysR and CTLD1 domains of PLA2R. This is consistent with prior serological studies identifying the N-terminal region of PLA2R as immunodominant in MN (18, 32, 34). Notably, we did not recover antibodies against more C-terminal domains such as CTLD7, which have been associated with epitope spreading in MN (32). This likely reflects both methodological and biological factors, including preferential isolation of high-affinity B cell clones, the early disease stage of our patient cohort, in which C-terminal reactivity may be less prevalent, or stochastic factors that may have contributed to the failure. These observations reinforce the concept that early humoral responses in MN are focused on the N-terminal PLA2R domains, which may be most relevant for initiating pathogenic immune complex formation.

A central finding of our study is that individual mAbs were largely insufficient to induce robust complement activation and lysis of PLA2R-expressing podocytes, even when expressed as complement-activating subclasses. Only a minority of IgG1-converted antibodies induced modest CDC, highlighting the notion that epitope specificity alone does not determine pathogenicity. Instead, we demonstrate that cobinding of antibodies to distinct epitopes — particularly across CysR and CTLD1 — synergistically enhanced complement activation. This effect was substantially greater than that observed with antibodies targeting the same domain, indicating that spatially distinct epitope engagement was required to efficiently cluster Fc regions and initiate the classical complement pathway. In addition, C1q engagement occurred through binding to clustered Fc regions of antigen-bound antibodies and was markedly enhanced when multiple IgG molecules were arranged in close proximity, facilitating efficient complement activation. This is consistent with our observation that pairing antibodies targeting distinct epitopes was required to achieve optimal CDC.

These findings provide a mechanistic framework for clinical observations linking broader epitope recognition with disease severity. Longitudinal studies have shown that patients with reactivity to multiple PLA2R domains exhibit more severe disease and worse outcomes (20, 32). Our data suggest that such epitope diversification was not merely a marker of disease progression but may have directly enhanced pathogenicity by enabling cooperative antibody interactions that promoted complement activation. Structural proximity between the CysR and CTLD1 domains likely facilitated this process, as supported by our epitope binning data demonstrating both competitive and noncompetitive binding patterns among mAbs.

Importantly, the relevance of epitope pairing extends beyond in vitro systems. In a human PLA2R-expressing mouse model, we observed that complement deposition and glomerular injury increased with the number and diversity of cobinding antibodies, with the strongest effects seen when both CysR- and CTLD1-reactive antibodies were present. Interestingly, there was a certain discrepancy between the strong increase in mIgG binding when different mAbs were paired and the less prominent increase in C3 deposition. In addition, while the combination of 2 mAbs induced maximum complement activation in vitro, the combination of 4 mAbs was necessary in mice to induce prominent glomerular complement deposition, and repetitive dosing was necessary to cause proteinuria. These effects could be the result of the action of different Fc parts (human Fc in vitro, mouse Fc in vivo), differences in the used complement components (rabbit complement in vitro, mouse complement in vivo), or distinct complement regulation mechanisms.

Sustained exposure to antibody combinations resulted in the development of albuminuria, supporting the concept that chronic immune complex formation involving multiple epitopes drives progressive podocyte injury (40). These findings are in accordance with studies in the MN rat model of Heymann nephritis, in which polyvalent IgG is necessary to induce the formation of larger subepithelial immune deposits (41). Our in vivo findings thus reinforce the idea that antibody diversity and epitope cooperation are key determinants of disease activity in MN.

Although IgG4 is the dominant anti-PLA2R subclass in patients (3), our results highlight a critical role for IgG1 and IgG3 as primary mediators of classical pathway activation. Both subclasses efficiently induced CDC when paired across epitopes, whereas IgG4 antibodies alone were incapable of activating complement in our system. These findings are consistent with the known inability of IgG4 to bind C1q and activate the classical pathway (42). However, the coexistence of IgG1 and IgG3 with IgG4 in patient samples suggests a more nuanced interplay between subclasses. Indeed, we demonstrate that IgG4 can modulate complement activation in a context-dependent manner. When combined with IgG1, IgG4 antibodies moderately enhanced CDC, probably by contributing to Fc clustering. Conversely, at higher concentrations or when targeting overlapping epitopes, IgG4 inhibited complement activation, consistent with competitive binding that disrupts optimal Fc arrangement by IgG1/IgG3. These dual effects suggest that IgG4 may act as a regulator of antibody-mediated effector functions, balancing pathogenic and protective roles depending on its relative abundance and epitope specificity.

Our findings also provide insight into the apparent paradox of limited C1q deposition in MN despite evidence for classical pathway activation. While C1q staining is often weak or absent in kidney biopsies when using cryoconserved tissue, prior studies using paraffin-embedded tissue and proximity ligation assays have demonstrated colocalization of IgG with C1q in patient samples (25). Furthermore, sera containing mixed IgG subclasses exhibit greater cytotoxic potential than IgG4-only sera (23). Our data mechanistically support these observations by showing that even low-abundance IgG1/IgG3 antibodies can drive substantial complement activation when appropriately paired across epitopes, thereby amplifying their pathogenic effect.

In addition to classical pathway activation, previous studies have implicated IgG4 in lectin pathway activation via mannose-binding lectin (MBL), potentially driven by altered Fc glycosylation (27). While the use of recombinant antibodies in our study does not take into account such a mechanism, the use of recombinant anti-PLA2R mAbs allowed us to elucidate the specific mechanism of anti-PLA2R epitope pairing and to reduce potential confounding by the diversely glycosylated polyclonal anti-PLA2R IgG in patient serum. Thus, distinct complement pathways may be engaged by different antibody subclasses in MN, with IgG1/IgG3 primarily activating the classical pathway and IgG4 potentially contributing via lectin pathway mechanisms.

Our study has several limitations. First, the mAbs used in this study were derived from only 4 patients and thus probably do not cover the full spectrum of the PLA2R-directed immune response in MN. Second, we did not explore the relationship between epitope pairing and epitope spreading over time, which would require longitudinal collection of serum samples. Such efforts are currently ongoing as part of international collaborations. Third, the roles of the lectin pathway, membrane-expressed and soluble complement regulatory proteins, and podocyte complement receptor signaling (such as by C3aR and C5aR) were not explored in our study and remain incompletely understood in the context of PLA2R-associated MN. In particular, future studies should look at the effect of C3aR- and C5aR-specific blockade and knockdown on readouts like cytoskeletal disruption, loss of nephrin or synaptopodin, and altered podocyte permeability or survival. Importantly, many of these factors may also substantially differ between humans and mice.

Finally, our study may have important therapeutic implications. Current treatments for MN primarily target B cells or broadly suppress the immune system, but are associated with delayed efficacy, relapse, and significant adverse effects (38, 43). Our data suggest that disruption of epitope pairing at the level of antigen-antibody interaction may represent a therapeutic strategy. We demonstrate that Fab fragments targeting either CysR or CTLD1 effectively inhibited CDC by patient-derived antibodies, supporting the concept that competitive blockade of key epitopes can prevent pathogenic antibody synergy. Such approaches could complement existing immunosuppressive therapies by providing more immediate protection against complement-mediated injury.

In conclusion, our study identifies epitope pairing as a fundamental mechanism driving complement activation in PLA2R-associated MN. Cooperative binding of antibodies to distinct domains enabled efficient Fc clustering and activation of the classical complement pathway, with IgG1 and IgG3 playing a central role despite their lower abundance. IgG4, though noncomplement activating on its own, modulated these processes in a context-dependent manner. These findings not only advance our understanding of MN pathogenesis but also highlight epitope-specific targeting strategies as a promising avenue for therapeutic intervention.

## Methods

### Sex as a biological variable.

Both male and female participants were included in the study to ensure representation of both sexes. Similarly, both male and female mice were included in the animal studies because the investigated mechanisms were not expected to be sex-specific. Findings are reported for both sexes.

### Study population and sample collection.

This prospective observational study aimed to assess the characteristics of anti-PLA2R autoantibodies in primary MN. The enrollment period spanned from August 2017 to July 2021, during which adult patients (≥20 years of age) with a histologic diagnosis of anti-PLA2R–associated MN were recruited. Clinical and laboratory data were systematically collected at the time of the biopsy, with a thorough review of clinical history and presentations conducted by the investigators. Plasma and blood cells from the participants were collected for studies.

### Cell culture conditions.

Conditionally immortalized human podocyte cell lines (AB8/13 and LY), provided by Moin Saleem (University of Bristol, Bristol, United Kingdom), were cultured as previously described (44). Human embryonic kidney (HEK) 293 and HEK293T cells (gifted by Ya-Fang Chiu Lab, Chang Gung University, Taoyuan, Taiwan) were cultured in DMEM supplemented with 10% FBS and 1% penicillin/streptomycin. Freestyle 293-F (Thermo Fisher Scientific) cells were cultured in serum-free FreeStyle 293 Expression Medium (Gibco, Thermo Fisher Scientific).

### Isolation of single human B cells by FACS.

Mononuclear cells were isolated from peripheral venous blood using Ficoll-Paque (GE Healthcare) density gradient centrifugation, following the manufacturer’s instructions. Purified mononuclear cells were resuspended in FACS buffer (1% FBS, 2 mM EDTA in PBS) containing 5% normal mouse serum (Jackson ImmunoResearch) at a concentration of 1 × 10^7^ cells/mL and incubated for 30 minutes on ice. Before sorting, cells were stained on ice for 30 minutes with anti–human CD3-PE-Cy7 (BioLegend, UCHT1, 300420), anti–human CD19-APC-Cy7 (BioLegend, HIB19, 302218), anti–human IgG-BV421 (BD Biosciences, G18-145, 562581), anti–human IgD-FITC (BD Biosciences, IA6-2, 555778), anti–Myc-APC (R&D Systems, 9E10, IC3696A), and 7-aminoactinomycin D (MilliporeSigma, A9400; 1 mg). Single B cells bound to PLA2R protein were identified using the following gating strategy: CD19^+^IgG^+^CD3^–^IgD^–^Myc^+^. These cells were sorted into individual wells of 96-well plates containing 18 μL/well RT-lysis buffer. The plates were sealed with aluminum sealing tape (Corning) and stored at –80°C.

### Single-cell reverse transcription PCR and Ig gene amplification.

Reverse transcription PCR (RT-PCR) was carried out manually. cDNA synthesis was performed at a volume of 20 μL/well in the original 96-well sorting plate. Total RNA from single cells was reverse-transcribed in diethyl pyrocarbonate-treated (DEPC-treated) water (Ambion) using 200 ng random hexamer primer (Thermo Scientific), 1 μL of 10 mM deoxynucleoside triphosphate (dNTP) mix (Thermo Scientific), 0.5% v/v Igepal CA-630 (MilliporeSigma), 40 U Ribolock (Fermentas), and 50 U Maxima H minus reverse transcriptase (Thermo Fisher Scientific). The reverse transcription reaction was conducted at 42°C for 10 minutes, 25°C for 10 minutes, 50°C for 45 minutes, and 85°C for 5 minutes. cDNA was stored at –20°C. IgH, Igκ, and Igλ V gene transcripts were independently amplified by nested PCR using 2.5 μL cDNA as a template. PCR reactions were carried out at a total volume of 25 μL of 0.5 μM primer mix, 200 μM (dNTP) (Thermo Fisher Scientific), and 0.5 U Phusion High-Fidelity DNA polymerase (Thermo Fisher Scientific) with an error rate of approximately 4.4 × 10^–7^. All primers were stored in aliquots to avoid repeated freezing and thawing. Nested PCR reactions with gene-specific primers or primer mixes were performed with 2.5 μL of the unpurified first PCR product. Each round of PCR was conducted for 35 cycles at 98°C for 10 seconds, 65°C for 15 seconds, and 72°C for 30 seconds.

### Ig gene sequence analysis.

The second PCR products of the VH, Vκ, and Vλ chains were purified using a QIAquick PCR purification kit (Qiagen) according to the manufacturer’s instructions and sequenced with the respective primer. Sequences were analyzed using IMGT®, the international ImMunoGeneTics (IMGT) information system (http://www.imgt.org) to identify germline V(D)J gene segments with the highest identity.

### IgG expression vector cloning.

Before constructing expression vectors, all PCR products were purified using the QIAquick PCR purification kit (Qiagen) and eluted with 25 μL DEPC-treated water (Ambion). After sequencing individual clones, gene-specific primers were selected according to the V or J segments with the highest similarity. PCR products were purified and recombined into human IgG1κ or IgG1λ expression vectors (provided by Tse-Wen Chang, Academia Sinica, Taipei, Taiwan) using the GeneArt Seamless Cloning and Assembly Enzyme Mix (Invitrogen, Thermo Fisher Scientific). Competent *E*. *coli* were transformed at 42°C with 5 μL of the recombination product. Colonies were screened by PCR using pIgG1κ-screen+ or pIgG1λ-screen+ as forward primers and pIgG1-screen– as the reverse primer. PCR products of the expected size (1,800 bp) were sequenced to confirm equivalent identity with the original PCR products. Plasmid DNA was isolated from 3 mL bacterial cultures grown for 16 hours at 37°C in Luria-Bertani broth containing 100 μg/mL ampicillin using QIAprep Spin columns (Qiagen).

To generate IgG3 and IgG4 versions, the Fc region of the pIgG1 plasmid was replaced with the IgG3 or IgG4 Fc sequence. The IgG Fc sequence was PCR-cloned from pVITRO1-trastuzumab-IgG3/κ or pVITRO1-trastuzumab-IgG4/κ (Addgene, 61885 and 61887). To enhance stability, mutations were introduced: R305H for IgG3 and R409K for IgG4. After modification, the Fc sequence of IgG3 or IgG4 was ligated into the pIgG1 vector using XhoI/NheI restriction digestion and T4 ligase.

### Cloning of soluble full-length and truncated PLA2R.

The DNA sequence for the extracellular region of wild-type human PLA2R, following the reference by Durga Kanigicherla et al., 2013 (gene ID: 22925), was cloned into 2 parts (A, covering nucleotides 60–1788, and B, covering nucleotides 1545–4191) using the pSectag2B transfer vector. After digestion with EcoRV and XhoI enzymes, the sequences were verified, and clones A and B were ligated and recloned into the pSectag2B vector (Supplemental Figure 1A). Wild-type human PLA2R cDNA was used as a template to construct truncated PLA2R domains. PCR fragments were generated, spanning from the CyRs domain to CTLD7. The truncated forms included: ΔF (deletion from Gln-36 to Thr-223), Δ1 (deletion from Gln-36 to Tyr-357), Δ2 (deletion from Gln-36 to Ala-504), Δ3 (deletion from Gln-36 to Pro-660), Δ4 (deletion from Gln-36 to Lys-805), Δ5 (deletion from Gln-36 to Lys-947), Δ6 (deletion from Gln-36 to His-1105), Δ7 (deletion from Gln-36 to Pro-1236), the CyRs domain (Gln-36 to Asp-165), and the CTLD1 domain (Asp-166 to Tyr-357). Each clone was produced using PCR and subsequently inserted into the pSectag2B plasmid using seamless cloning. All constructs were verified by DNA sequencing.

### Western blotting.

PLA2R protein was mixed with 4× dye buffer (Bio-Rad) without β2-ME and without heat treatment and then resolved on 8% SDS-PAGE and transferred onto 0.45 μm PVDF membranes (Merck). The membrane was probed with anti-PLA2R antibody (MilliporeSigma, polyclonal, HPA012657) at 1:5,000 dilution in blocking buffer (5% nonfat dry milk with 0.1% Tween 20). After washing with TBS-T, the membrane was incubated with HRP-conjugated rabbit anti-mIgG (Jackson ImmunoResearch, 315-035-044) at a 1:5,000 dilution and visualized using ECL reagent (Merck). Cells were washed with PBS and lysed using protein extraction buffer supplemented with Halt protease inhibitor cocktail (Thermo Fisher Scientific). Lysates were centrifuged at 16,000*g* for 15 minutes at 4°C to collect precleared cell extracts. The PVDF membrane was blocked with 5% nonfat milk in blocking buffer for 1 hour, followed by incubation with patient serum diluted 1:100 overnight at 4°C. The membrane was washed and incubated with HRP-conjugated mouse anti–human IgG (Jackson ImmunoResearch, 209-035-088) at a 1:5,000 dilution for 1 hour, after which ECL detection was performed.

### Lentiviral infection system.

The full-length human *mPLA2R* gene was subcloned into the pLJM-EGFP lentiviral expression vector (Addgene, 19319) with an N-terminal EGFP tag. HEK293 cells at 70%–80% confluence were transfected with the pLJM-mPLA2R-EGFP vector, along with the packaging plasmid pCMV-delta R-8.91 (Academia Sinica) and the envelope plasmid pCMV-VSVG (Academia Sinica) at a 10:9:1 ratio, using the T-PRO NTR2 transfection reagent (T-Pro Biotechnology) in accordance with the manufacturer’s protocol. Podocytes (AB8/13 and LY) at the permissive stage were infected with mPLA2R lentiviral particles in the presence of 8 μg/mL polybrene for 18 hours, after which the medium was replaced. Infected cells were used in subsequent experiments, 48 hours after lentiviral infection.

### BLI analysis.

Antibody-binding kinetic rate constants, including association (*k_a_*) and dissociation (*k_d_*) rates, were measured using BLI on the ForteBio Octet RED96 system. The assay was performed with anti-hIgG Fc capture (AHC) biosensors (ForteBio) to capture anti-PLA2R mAbs at a concentration of 750 ng/mL, achieving a 0.5 nm shift. The biosensors were then immersed in solutions containing varying concentrations (0, 3.125, 6.25, 12.5, 25, 50, 100, and 125 nM) of recombinant soluble PLA2R protein. The running buffer consisted of 0.1% BSA, 0.1% Tween 20, 250 mM NaCl, 2.7 mM KCl, 10 mM Na_2_HPO_4_, and 1.8 mM KH_2_PO_4_ in sterile water. Rate constants were determined by curve fitting analysis (using a 1:1 Langmuir binding model) of the binding response during a 5-minute association phase and a 15-minute dissociation phase.

### Sandwich epitope binning.

BLI was also used to perform epitope binning on a set of anti-PLA2R mAbs. Initially, the primary anti-PLA2R mAb was loaded onto AHC biosensors to obtain a fixed response. Following 100-second and 120-second baseline steps, soluble PLA2R was loaded in a dose-dependent manner within a 600-second association step. Subsequently, a second anti-PLA2R mAb was introduced for a 600-second incubation period on the biosensors. If signal accumulation was observed, it was indicative of binding to a different epitope; otherwise, binding to the same epitope was inferred.

### ELISA.

A polystyrene 96-well, flat-bottomed plate (Thermo Fisher Scientific) was coated with purified sPLA2R, the CysR domain, or the CTLD1 domain in sodium bicarbonate buffer (pH 9.6) for 18 hours. To measure the total anti-PLA2R antibody titer, 62.5 ng purified sPLA2R was used and showed an efficiency comparable to that of the commercial EUROIMMUN kit in detecting anti-PLA2R antibodies (Supplemental Figure 1C). For epitope mapping, 300 ng of either purified CysR or CTLD1 domains was coated onto the plate. After coating, the plates were blocked with 5% human albumin for 1 hour. Patients’ plasma was diluted to 1:100, or anti-PLA2R mAbs (1 μg/mL) were added in 5% human albumin. After a 1-hour incubation at room temperature (RT) on a plate shaker, plates were washed 3 times with PBS containing 0.1% Tween 20. A 100 μL volume of the appropriate HRP-conjugated secondary antibody, diluted in 5% human albumin, was added to each well and incubated for 1 hour. Total human IgG was detected using HRP-conjugated mouse anti–human IgG Fcγ fragment–specific antibody (Jackson ImmunoResearch, 209-035-098; 1:2,500). IgG subclass–specific antibodies were mouse monoclonal anti–human IgG1 hinge heavy-chain–HRP (Abcam, 4E3, ab99774; 1:2,500), mouse monoclonal anti–human IgG2 Fc-HRP (Abcam, 31-7-4, ab99779, 1:2,500), mouse anti–human IgG3 hinge-HRP (SouthernBiotech, 9210-05, 1:2,000), and mouse monoclonal anti–human IgG4 pFc′-HRP (Abcam, HP6023, ab99817; 1:2,500). After washing, the enzyme substrate TMB (MilliporeSigma) was added, with differing developmental times for each subclass (IgG: 9 min, IgG1: 35 min, IgG2: 20 min, IgG3: 60 min, IgG4: 5 min 30 s). The reaction was stopped with 0.5 mol/L H_2_SO_4_. Absorbance was measured at 450 nm using a PerkinElmer plate reader, and values were assigned to samples.

### CDC assay.

The CDC activity of anti-PLA2R mAbs was assessed using the CellTiter-Glo Luminescent Cell Viability Assay (Promega). PLA2R-overexpressing podocytes were harvested and resuspended in RPMI 1640 medium supplemented with 10% FBS. A total of 5,000 cells per well were seeded in a 96-well, flat-bottomed white plate and incubated overnight. The next day, the medium was replaced with RPMI containing 2% FBS (100 μL per well), and either mAbs (6.6 nM) or patient-derived IgGs (different IgG percentages were used in each assay: 50% in Figure 7A, 2.5% in Figure 7C, and 15% in Figure 7, D and E, and Figure 8) were added and followed by a 1-hour incubation at 37°C. Subsequently, 5% baby rabbit complement (Cedarlane) was added to each well, and the cells were incubated for 4 hours at 37°C. After incubation, 100 μL CellTiter-Glo reagent was added, and the plates were incubated for 15 minutes at RT on a shaker (70 rpm). Luminescence was measured using a PerkinElmer plate reader. To evaluate competitive binding, cells were pretreated with Fab fragments (132 nM) or PLA2R domains (132 nM) targeting either the CysR or CTLD1 domain for 1 hour at 37°C before treatment with mAbs or patient IgGs. For experiments using heat-inactivated (HI) serum, baby rabbit complement was heat inactivated at 56°C for 30 minutes prior to use. Fab fragments were generated using the Pierce Fab Micro Preparation Kit (Thermo Fisher Scientific), and MN patient IgGs were isolated using protein G mag Sepharose (Cytiva) as described in the immunoprecipitation section below.

Antibody-specific CDC was calculated using the following formula: % viability = 100 × *E/S*, % cytotoxicity = 100 × (1 – *E/S*), where *E* is the luminescence with experimental antibody, and *S* is the luminescence without antibody, but with the same concentration of serum.

### Immunoprecipitation IgGs from patient plasma.

To evaluate the ability of total IgG from patients with MN to activate CDC in mPLA2R-podocytes, 300 μL patient plasma was mixed with 150 μL protein mag Sepharose (Cytiva) in a total volume of 1 mL PBS to capture patient IgGs. The mixture was incubated overnight with constant rotation at 4°C. After incubation, the resin was washed 3 times with PBS. Protein elution was carried out by incubating the resin with 300 μL of 0.1 M glycine for 10 minutes at RT. Following this, 30 μL of 1 M Tris buffer was added to neutralize the solution. The IgGs were dialyzed in a dialysis membrane against PBS for 3 days. Finally, the IgGs were filtered through a 0.22 μm membrane filter to remove impurities.

### Animal experiments.

Human PLA2R1 transgenic mice were generated as previously described (16) and bred and maintained at the University Medical Center Hamburg-Eppendorf in a specific pathogen–free facility at temperatures of 21°C–24°C with 40%–70% humidity on a 12-hour light/12-hour dark cycle and provided with food and water ad libitum.

For passive transfer experiments, mice received control (R&D Systems, 133304, MAB00321) or anti-PLA2R mAb (both mIgG2a) i.p. The first cohort thus involved 4 groups of mice receiving anti-PLA2R mAbs: 1 mAb (2B8, 1B7, 1B10, 1D1; 100 μg each), 3 groups of mice receiving 2 mAbs (2B7/1B7, 2B8/1B10 and 1B10/1D1; 50 μg each), 2 groups receiving 3 mAbs (2B8/1B7/1B10 and 2B8/1B10/1D1; 33 μg each), and 1 group receiving 4 mAbs (2B8/1B7/1B10/1D1, 25 μg each). Each group included 3 animals. Mice were bled 2 days after injection to measure anti-PLA2R levels. Mice were euthanized by perfusion 10 days after mAb injection. The second cohort involved 1 group receiving anti-PLA2R mAbs (4 mAbs: 2B8/1B7/1B10/1D1; 250 μg each). Animals were bled 10 days after injection to measure anti-PLA2R levels, received a second mAb dose at day 15, and were euthanized 30 days after the first injection.

### Immunofluorescence analysis of mouse kidney tissue.

Paraffin-embedded kidney sections (3 μm) from experimental mice were deparaffinized and rehydrated to water for immunofluorescence staining. Antigen retrieval was carried out either by heat-mediated retrieval in citrate buffer (pH 6.1, 98°C for 30–60 min) or by enzymatic digestion with protease XXIV (5 μg/mL; MilliporeSigma) for 15 minutes at 37°C. After blocking nonspecific binding with 5% horse serum (Vector Laboratories) supplemented with 0.05% Triton X-100 in PBS for 30 minutes at RT, sections were incubated overnight at 4°C with primary antibodies diluted in blocking buffer consisting of C3 (Abcam, ab200999, 1:500), WGA-rhodamine (1:400; Vector Laboratories, RL-1022), and laminin (1:1,000; MilliporeSigma, L9393). Staining was detected using fluorophore-conjugated Cy2 donkey anti–mIgG H+L (1:200; Jackson ImmunoResearch, 715-225-150), and Cy3 donkey anti–rabbit IgG H+L (1:200; Jackson ImmunoResearch, 711-165-152) for 30 minutes at RT. Nuclei were counterstained with Draq5 (1:1,000; Cell Signaling Technology). Images were acquired on a Zeiss LSM800 Airyscan confocal microscope using ZEN blue software.

### Anti-PLA2R detection in mouse serum by ELISA.

High-binding 96-well ELISA plates (Sarstedt) were coated overnight at 4°C with the extracellular region of human PLA2R1 (hPLA2R1) diluted in carbonate-bicarbonate coating buffer (C3041, MilliporeSigma; 100 ng/well). Plates were washed 3 times with wash buffer (TBS containing Tween 20, T9039, MilliporeSigma) and blocked with 100 μL/well postcoat buffer (TBS containing BSA, T6789, MilliporeSigma) for 30 minutes at RT. After 3 additional washes, mouse sera diluted 1:100 in postcoat buffer supplemented with 0.05% Tween 20 were added (100 μL/well) and incubated for 1 hour at RT.

Plates were washed 3 times and incubated with HRP-conjugated anti–mIgG secondary antibody (1:10,000; Jackson ImmunoResearch, 715-035-151) for 1 hour at RT. Following washing, TMB substrate (Avia Systems Biology) was added for 2 minutes at RT, and the reaction was stopped with 100 μL of 1 mol/L H_3_PO_4_. Absorbance at 450 nm was measured using an ELISA reader (EL808, BioTek Instruments).

### Electron microscopic analysis in mice.

For electron microscopy, small pieces (2 mm^3^) of mouse renal cortex (*n* = 3 per condition) were immersion fixed in 4% paraformaldehyde in 0.1 M phosphate buffer (48 hours at 4°C). Tissue blocks were washed in 0.1 M phosphate buffer, treated with OsO_4_ (0.5% for 45 minutes at RT) and stained with uranyl acetate (1% w/v in 70% v/v Ethanol, 45 minutes at RT). After dehydration, tissue blocks were embedded in epoxy resin (Durcupan ACM, MilliporeSigma). Ultrathin sections (70 nm) were cut using an UC6 ultramicrotome (Leica) and analyzed with an 80 kV Zeiss LEO910 transmission electron microscope and Image SP software (TRS-Tröndle).

### Statistics.

Statistical analyses were performed using GraphPad Prism (GraphPad Software). Data are presented as scatter dot plots, with bars indicating the mean ± SD or mean ± SEM. Differences between 2 groups were analyzed using the Mann-Whitney *U* test or a 2-tailed, unpaired Student’s *t* test, as appropriate. Comparisons among multiple groups were analyzed using 1-way ANOVA followed by Tukey’s multiple-comparison test, as appropriate. Comparisons involving 2 independent variables were analyzed using 2-way ANOVA followed by Dunnett’s or Tukey’s multiple-comparison test, as appropriate. No samples were excluded from the analyses. A *P* value of less than 0.05 was considered statistically significant.

### Study approval.

These studies were approved by the local ethics committee of the IRB of Chang Gung Memorial Hospital (IRB nos. 201601889B0, 201802349B0, and 201902178B0 and 202002593B0) and were conducted in accordance with the ethics principles stated by the Declaration of Helsinki. All participants provided written informed consent. Animal experiments were performed according to national and institutional animal care and ethics guidelines and were approved by the Veterinarian Agency of Hamburg and the local animal care committee, Behörde für Justiz und Verbraucherschutz, Amt für Verbraucherschutz, Abteilung Lebensmittelsicherheit und Venterinärwesen (registration nos. 097/2023 and 040/2024).

Additional details on methods are provided in the Supplemental Methods.

### Data availability.

All data supporting the findings of this study are included in the main article, the supplemental material, and the Supporting Data Values file provided with this article, including uncropped gels and blots.

## Manuscript information

### Author contributions.

TYW contributed to study conceptualization, formal analysis, project administration, visualization, experiments, methodology, and validation. KHT supervised the study, contributed to study conceptualization, acquired funding, provided resources, wrote the original draft of the manuscript, and reviewed and edited the manuscript. LS performed animal experiments, analysis, and designed study methodology. KWL performed experiments, designed methodology, and validated results. HPS contributed to study conceptualization. JJP provided resources and reviewed and edited the manuscript. YFL contributed to study conceptualization. GZ generated essential tools and planned experiments. OK contributed to study methodology. YNL performed experiments, designed study methodology, and validated results. CXK, JYD, and CCL contributed to study conceptualization. YRT, LYM, YTC, and CWY provided resources. YHT reviewed and edited the manuscript. NMT supervised the work, contributed to study conceptualization, acquired funding, and reviewed and edited the manuscript. CLK supervised the work, contributed to study conceptualization, acquired funding, wrote the original draft and reviewed and edited the manuscript.

### AI disclosure.

ChatGPT 5.5 (OpenAI) was used to assist with language editing and refinement on May 20, 2026. No AI tools were used for data analysis, interpretation of results, or generation of scientific conclusions. The authors verified the accuracy of all manuscript content, approved the final manuscript, and take full responsibility for its content.

### Conflict of interest.

CLK is a cofounder, director, and employee of Elixiron. TYW, KHT, and CLK are submitting a patent application related to the current work, titled Anti-PLA2R antibodies and uses thereof. KHT reports serving as a national scientific partner for the AUTUMN clinical trial sponsored by Alexion, AstraZeneca Rare Disease. NMT is a cofounder of Global Immune, reports a patent for the measurement of anti-THSD7A antibodies (patent no. EP2015/066881), receives consultancy fees and research funding from Merida Bioscience, owns stocks of Merida Bioscience, and receives honoraria from CSL Vifor, StreamedUp, Novartis, AstraZeneca, and Boehringer Ingelheim.

### Funding.

This work was supported by the following:

- National Science and Technology Council of Taiwan (MOST 106-2314-B-182A-126, MOST 108-2314-B-182A-026, and MOST 109-2314-B-182A-119, to CLK).
- Chang Gung Memorial Hospital (BMRPB98, to CLK).
- German Research Foundation (DFG) through the “Initiation of International Collaboration” program (TO 1013), the CRC TRR 422, and the CRC 1192, to NMT).
- European Research Council (AUTO-TARGET, to NMT).
- Else Kröner-Fresenius-Stiftung through a Clinician Scientist Professorship (to NMT).

## Supplementary Material

Supplemental data

Unedited blot and gel images

Supporting data values

## Figures and Tables

**Figure 1:**
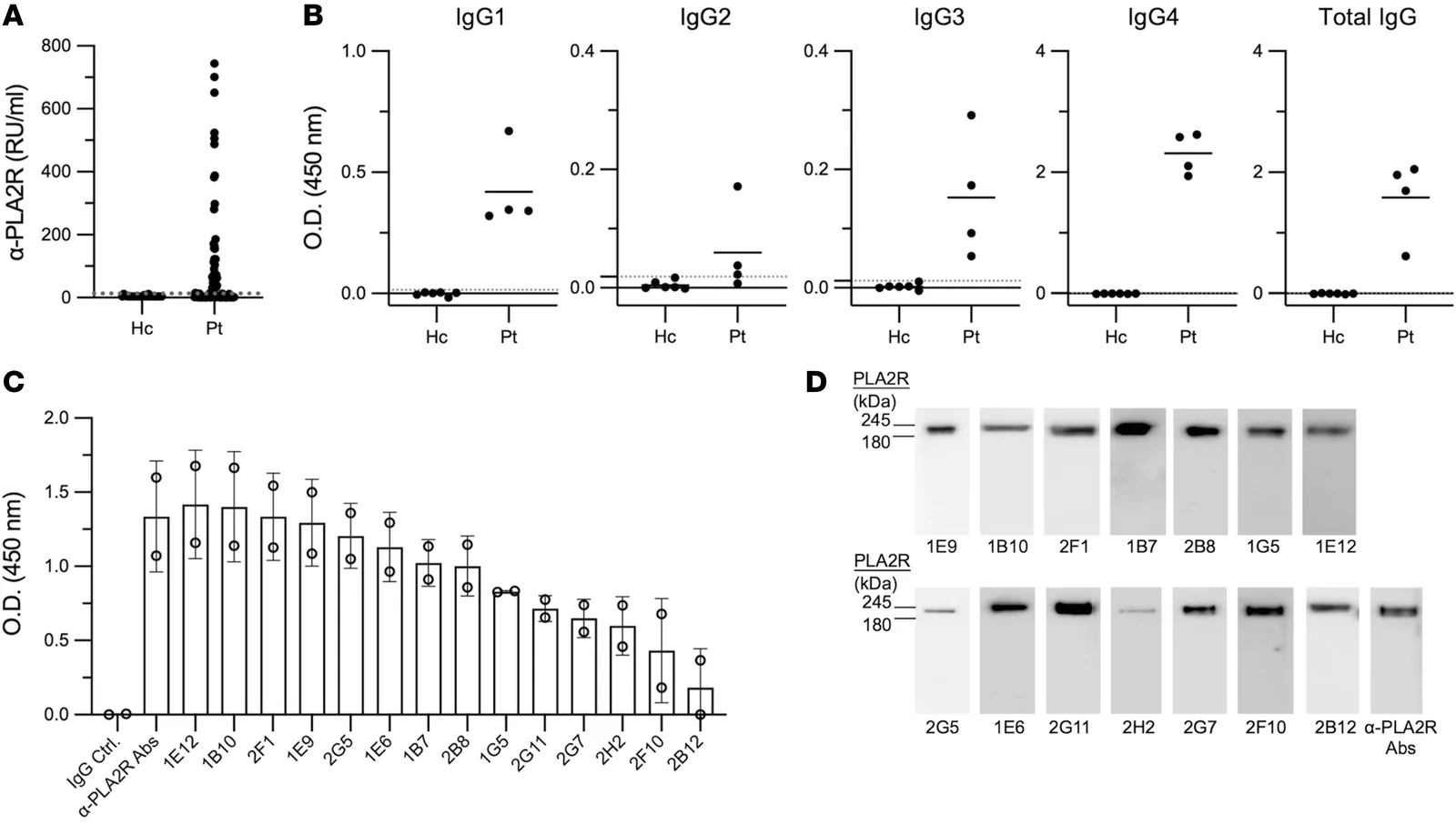
Generation of anti-PLA2R mAbs from peripheral blood of patients with PLA2R-associated MN. (**A**) Anti-PLA2R antibody levels as measured by ELISA in healthy individuals (Hc) and patients with PLA2R-associated MN (Pt). The dotted line indicates the cutoff for positivity (13 RU/mL) using the ELISA kit. (**B**) IgG subclass–specific anti-PLA2R antibody levels as measured by ELISA in the 4 patients with MN (Pt) who had the highest titers according to the data in **A**. The cutoff (dotted line) represents the mean plus 3 SDs of the levels measured in healthy individuals (Hc). Data are presented as the mean. (**C**) Reactivity of polyclonal anti-PLA2R antibodies (anti-PLA2R [α-PLA2R Abs], 1 μg/mL) and 14 anti-PLA2R mAbs with recombinant PLA2R (645 ng/mL) as measured by ELISA. Data are presented as the mean ± SD for 2 independent experiments. (**D**) Western blot showing the reactivity of 14 anti-PLA2R mAbs and polyclonal anti-PLA2R antibodies (α-PLA2R Abs, 1 μg/mL) with recombinant PLA2R (100 ng/well). The experiment was performed twice independently. MN, membranous neuropathy.

**Figure 2:**
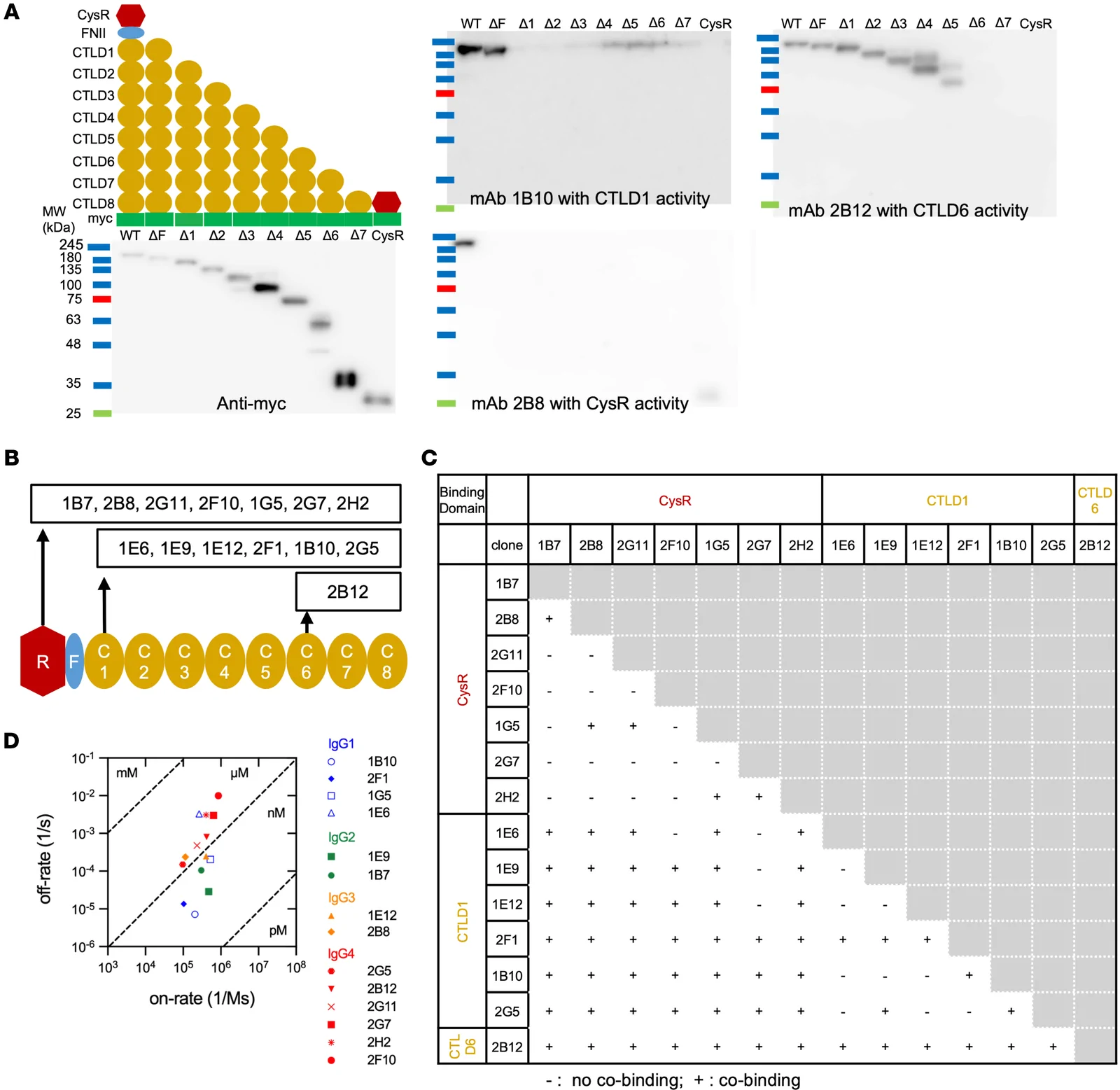
Binding characteristics of the anti-PLA2R mAbs. (**A**) Scheme of the PLA2R structure and the PLA2R mutants that were used for the identification of the epitope areas targeted by the patient-derived mAbs (upper panel). Representative Western blot analyses of recombinant PLA2R and PLA2R mutants with anti-PLA2R mAbs. The results for 3 different mAbs (targeting CysR, CTLD1, and CTLD6) are shown (lower panel). MW, molecular weight. (**B**) Schematic illustration summarizing the binding sites of the 14 isolated mAbs. (**C**) Results of epitope binning experiments using BLI analysis. “–” indicates the absence of cobinding to PLA2R, suggesting competition of the 2 clones; “+” indicates the presence of cobinding to PLA2R. (**D**) Scatter plot of equilibrium *K_D_* values of the 14 anti-PLA2R mAbs derived from the BLI experiments shown in Table 2. *K_D_* values were determined on the basis of the dissociation (off-rate, *y* axis) and association (on-rate, *x* axis) of the mAbs.

**Figure 3:**
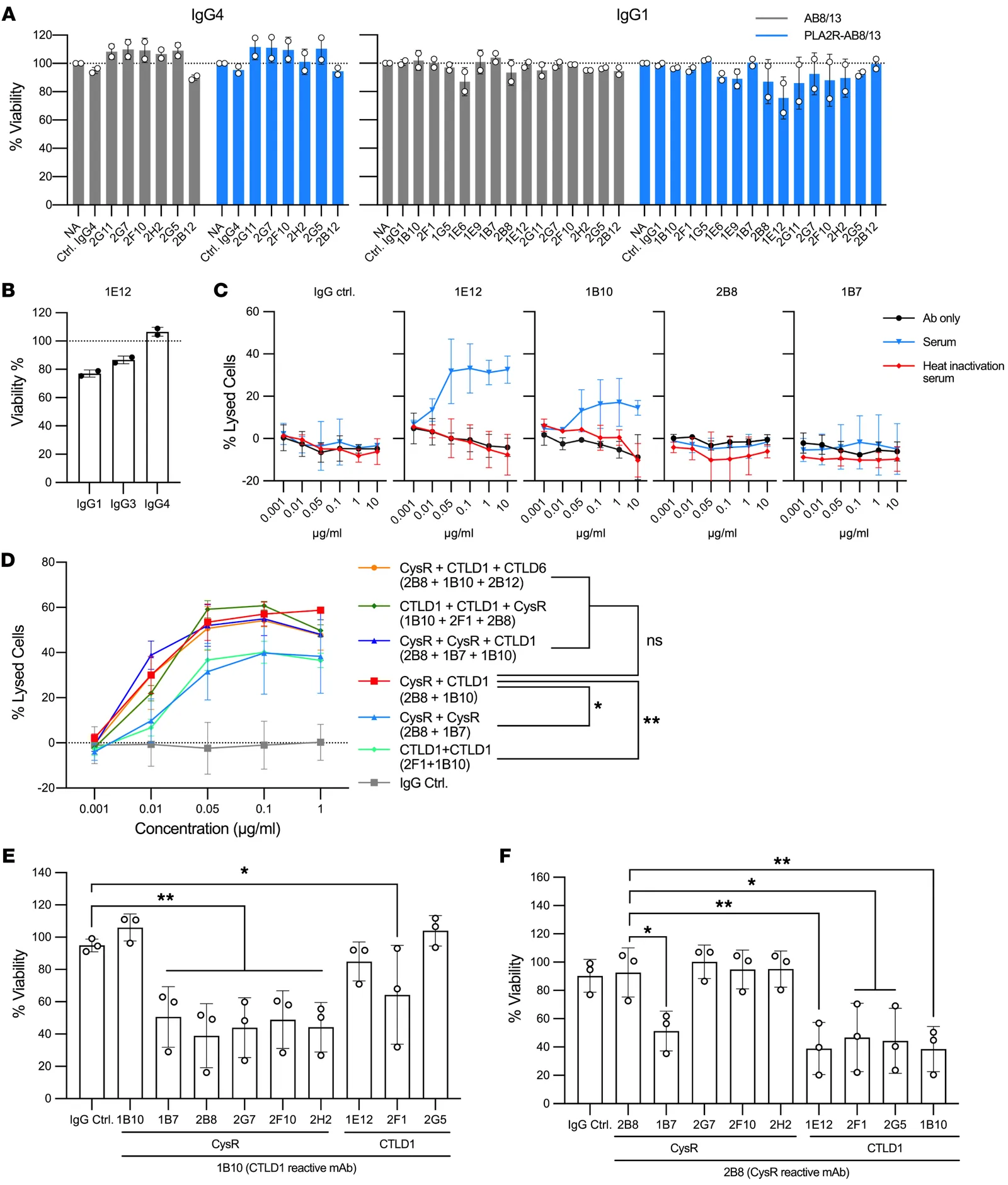
CDC induction by anti-PLA2R mAbs using PLA2R-expressing podocytes as target cells. (**A**) CDC assays using individual anti-PLA2R mAbs of IgG4 and IgG1 subclasses (antibody concentration: 10 μg/mL). Experiments were performed using both the untransduced parental podocyte line (AB8/13, gray) and the PLA2R-expressing podocyte line (PLA2R-AB8/13). (**B**) CDC assay comparing the 1E12 mAb clone of IgG1 with IgG4. (**C**) CDC assays using individual anti-PLA2R mAbs at different concentrations. Experiments were performed without serum as well as with heat-inactivated serum as negative controls. (**D**) CDC assays using different combinations of anti-PLA2R mAbs. (**E**) CDC assays using the CTLD1-reactive mAb (1B10 mAb, 1 μg/mL) paired with the other mAbs. (**F**) CDC assays using the CysR-reactive mAb (2B8 mAb, 1 μg/mL) paired with the other mAbs. Results represent the mean ± SD. *P < 0.05 and **P < 0.01, by 2-way ANOVA followed by Tukey’s multiple-comparison test (**D**) or 2-tailed, unpaired Student’s *t* test (**E** and **F**). Ctrl., control. *P < 0.05 and **P < 0.01.

**Figure 4:**
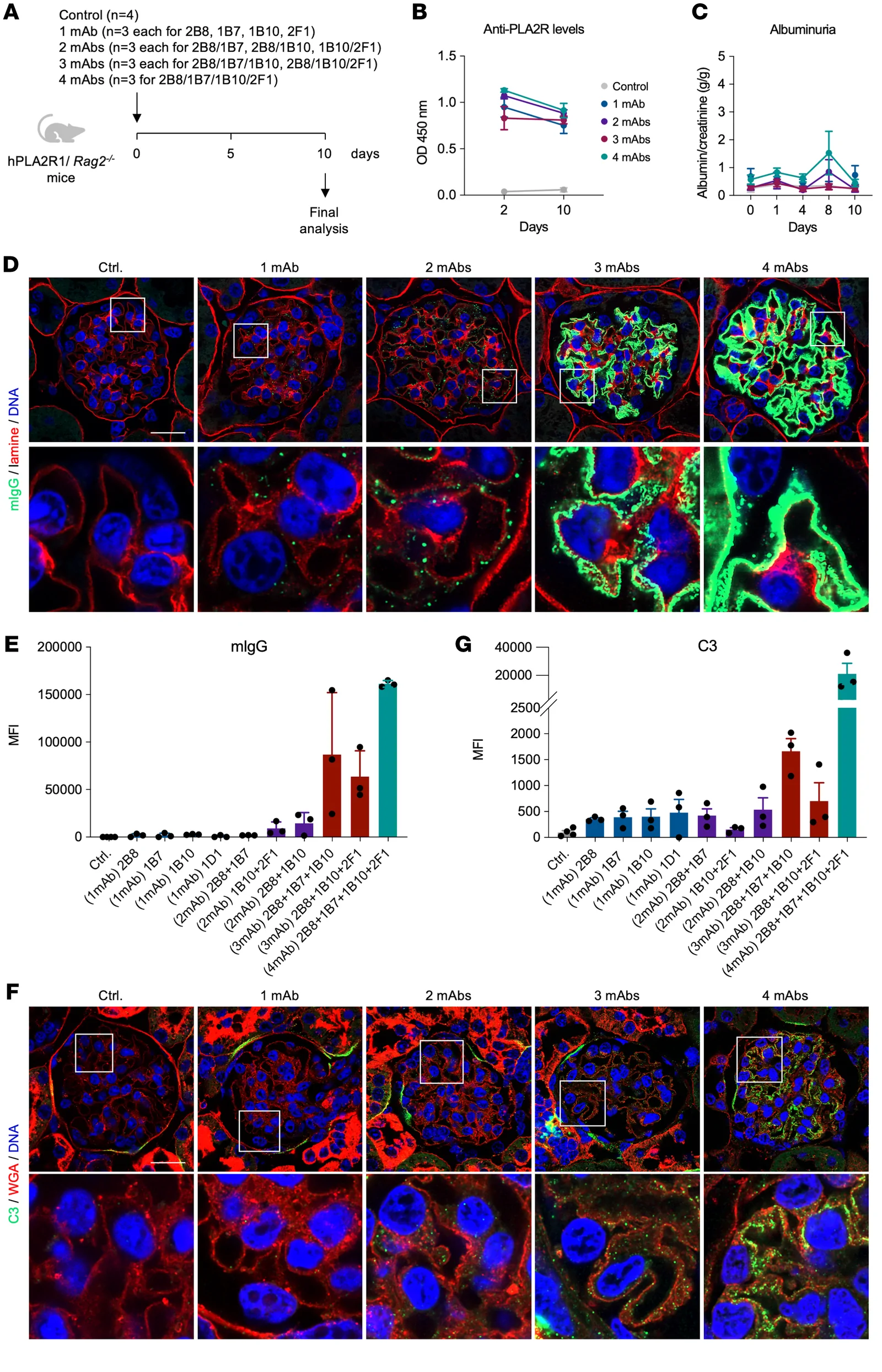
Epitope pairing of anti-PLA2R mAbs enhances complement deposition in vivo. (**A**) Schematic of the antibody transfer experiment and injection regimen in hPLA2R1-transgenic *Rag2^–/–^* mice. (**B**) Serum anti-PLA2R levels as measured by ELISA on days 2 and 10. (**C**) Albuminuria was measured by the urinary albumin/creatinine ratio after antibody transfer. (**D**) Representative immunofluorescence images of glomeruli from hPLA2R1-transgenic *Rag2^–/–^* mice 10 days after antibody transfer showing mIgG costained with the GBM marker laminin. Lower panels represent enlargements of the boxed areas in the upper panels. Scale bar: 20 μm. Original magnification ×63; insets ×5 relative to the original images. (**E**) Quantification of glomerular mIgG deposition (MFI) in hPLA2R1-transgenic *Rag2^–/–^* mice 10 days after antibody transfer. (**F**) Representative immunofluorescence images of glomeruli from hPLA2R1-transgenic *Rag2^–/–^* mice 10 days after antibody transfer showing C3 costained with wheat germ agglutinin (WGA). Lower panels represent enlargements of the boxed areas in the upper panels. Scale bar: 20 μm. (**G**) Quantification of glomerular C3 deposition (MFI) 10 days after antibody transfer. Data in **E** and **G** are shown as the mean ± SEM.

**Figure 5:**
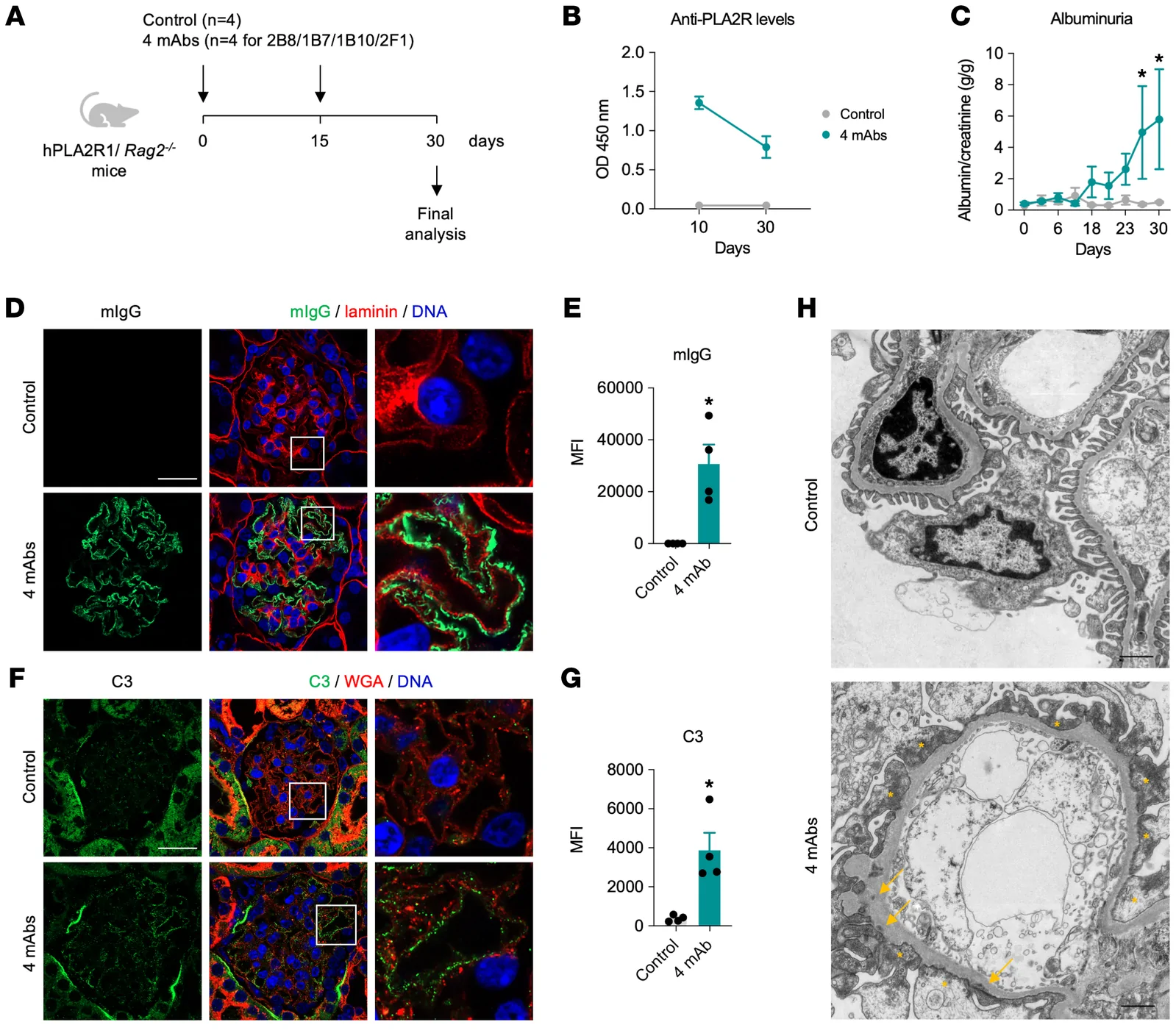
Epitope pairing by 4 anti-PLA2R mAbs enhances complement activation and proteinuria in vivo. (**A**) Schematic of the antibody transfer experiment and injection regimen in hPLA2R1-transgenic *Rag2^–/–^* mice. (**B**) Serum anti-PLA2R levels were measured by ELISA on days 11 and 30. (**C**) Albuminuria as measured by the urinary albumin/creatinine ratio after antibody transfer. (**D**) Representative immunofluorescence images of glomeruli from hPLA2R1-transgenic *Rag2^–/–^* mice 30 days after antibody transfer showing mIgG costained with the GBM marker laminin. Lower panels represent enlargements of the boxed areas in the upper panels. Scale bar: 20 μm. Original magnification ×63; insets ×5 relative to the original images. (**E**) Quantification of glomerular mIgG deposition (MFI) in hPLA2R1-transgenic *Rag2^–/–^* mice 30 days after antibody transfer. (**F**) Representative immunofluorescence images of glomeruli from hPLA2R1-transgenic *Rag2^–/–^* mice 30 days after antibody transfer showing C3 costained with WGA. Lower panels represent enlargements of the boxed areas in the upper panels. Scale bar: 20 μm. (**G**) Quantification of glomerular C3 deposition (MFI) 30 days after antibody transfer. (**H**) Electron microscopic analysis of the glomerular filtration barrier in hPLA2R1-transgenic *Rag2^–/–^* mice 30 days after antibody transfer. Asterisks indicate podocyte foot process broadening, arrow point at electron dense deposits. Scale bars: 1 μm. Data are shown as the mean ± SEM. *P < 0.05, by 2-way ANOVA (**C**) and 2-tailed Mann-Whitney *U* test (**E** and **G**).

**Figure 6:**
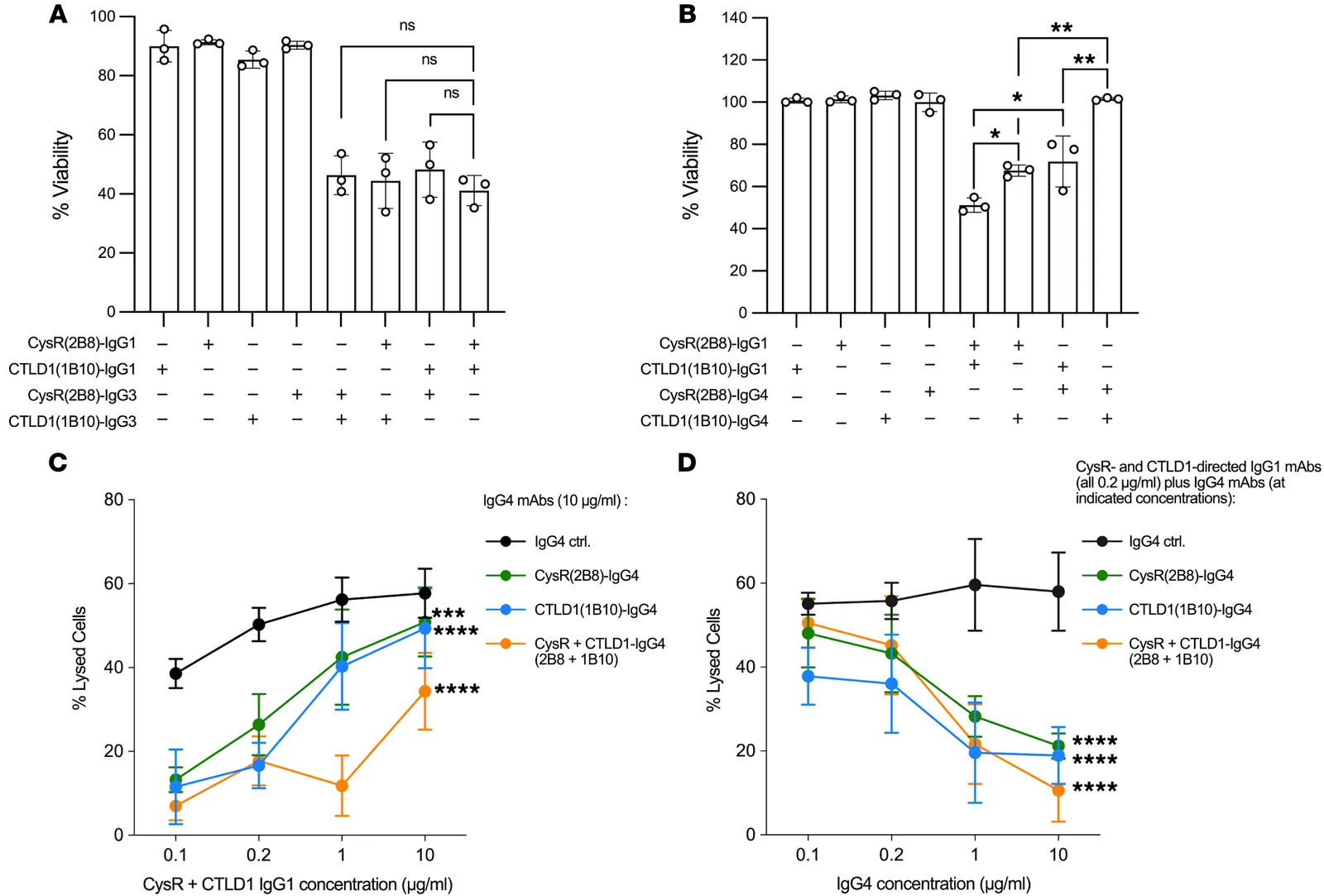
CDC activation by different anti-PLA2R mAb IgG subclasses. (**A**) CDC assays using a CTLD1-reactive mAb (1B10 mAb, 1 μg/mL) and a CysR-reactive mAb (2B8 mAb, 1 μg/mL) on IgG1 and IgG3 Fc backbones. (**B**) CDC assays using a CTLD1-reactive mAb (1B10 mAb, 1 μg/mL) and a CysR-reactive mAb (2B8 mAb, 1 μg/mL) on IgG1 and IgG4 Fc backbones. (**C**) CDC assays using different concentrations of combined CysR- and CTLD1-reactive IgG1 mAbs in the presence of control and anti–PLA2R IgG4 mAbs (at a fixed concentration of 10 μg/mL). (**D**) CDC assays using different concentrations of control and anti–PLA2R IgG4 mAbs in the presence of combined CysR- and CTLD1-reactive IgG1 mAbs (at a fixed concentration of 0.2 μg/mL). Data are shown as the mean ± SD. *P < 0.05, **P < 0.01, ***P < 0.001, and ****P < 0.0001, by 1-way ANOVA followed by Dunnett’s multiple-comparison test (**A** and **B**) or 2-way ANOVA followed by Dunnett’s multiple-comparison test (**C** and **D**). Asterisks in **C** and **D** indicate the comparison with the IgG4 control group.

**Figure 7:**
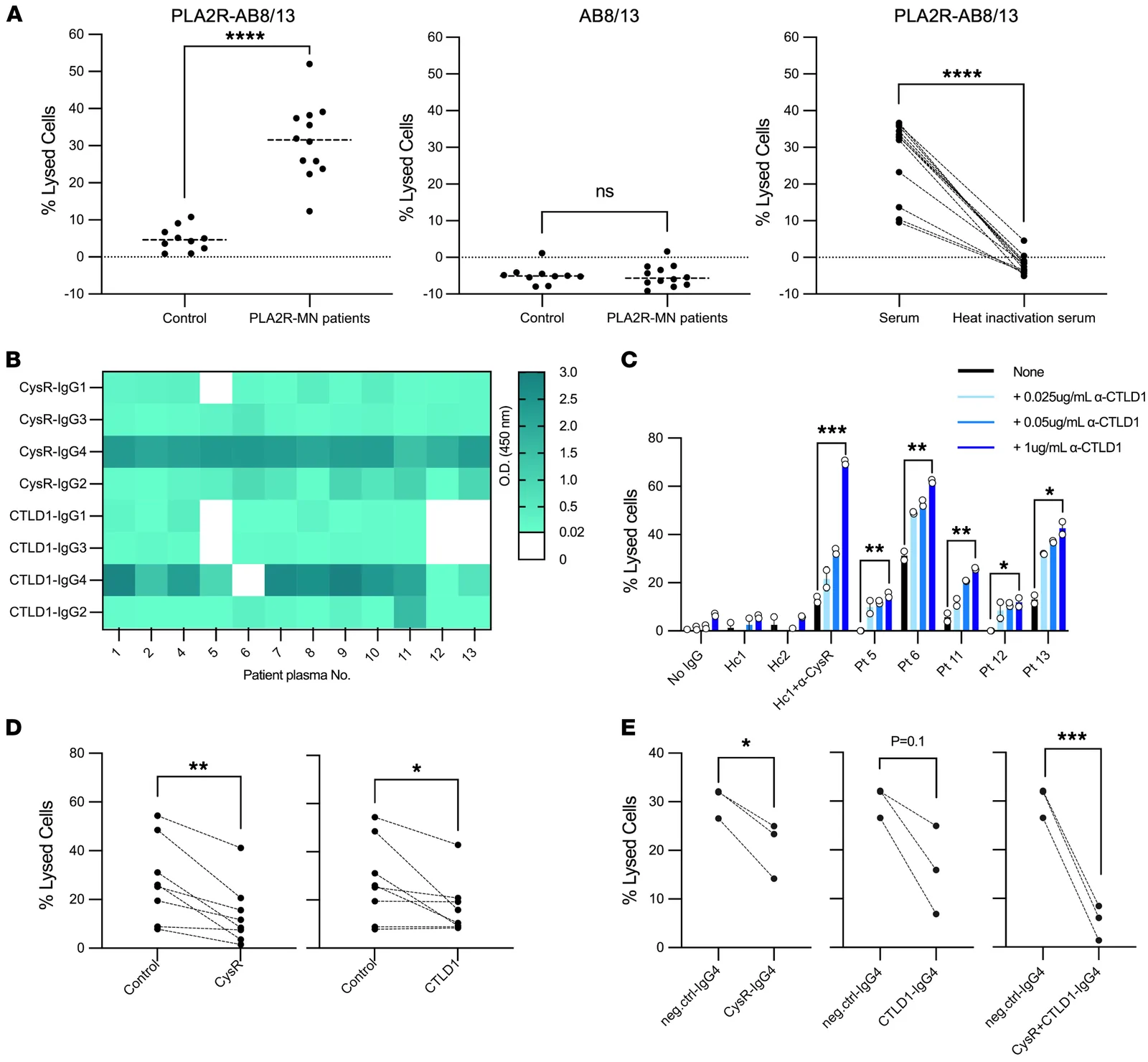
Epitope pairing and synergistic CDC activation by purified IgG from patients with PLA2R-associated membranous neuropathy. (**A**) CDC assays using the total IgG fraction from healthy individuals (control) and from patients with PLA2R-associated membranous neuropathy (MN) on PLA2R-expressing podocytes (PLA2R-AB8/13, left) and PLA2R-negative control podocytes (AB8/13, middle), as well as CDC assays using the total IgG fraction from patients with PLA2R-associated MN on PLA2R-expressing podocytes (PLA2R-AB8/13) using heat-inactivated serum as a source of complement (right). Data are presented as the mean. (**B**) Heatmap showing CysR- and CTLD1-specific reactivity of IgG1-4 present in the serum of patients with PLA2R-associated MN. An OD at 450 nm of 0.02 was set as the cutoff for positivity in this assay. (**C**) CDC assays using control and patient IgG with or without addition of different amounts of the CTLD1-reactive mAb (1B10). Controls included the absence of patient IgG, IgG from 2 healthy individuals, and IgG from one healthy individual in combination with an anti-CysR mAb (2B8). (**D**) CDC assays using patient IgG in the absence (control) or presence of recombinant CysR and CTLD1. (**E**) CDC assays using patient IgG in the presence of either a control IgG4 or anti-PLA2R IgG4 (10 μg/mL). Anti-PLA2R IgG4 included anti-CysR (2B8, left), anti-CTLD1 (1B10, middle), or a combination of both (right). neg.ctrl., negative control. *P < 0.05, **P < 0.01, ***P < 0.001, and ****P < 0.0001, by 2-tailed, paired Student’s *t* test (**D** and **E**) and 2-tailed, unpaired Student’s *t* test (**A** and **C**).

**Figure 8:**
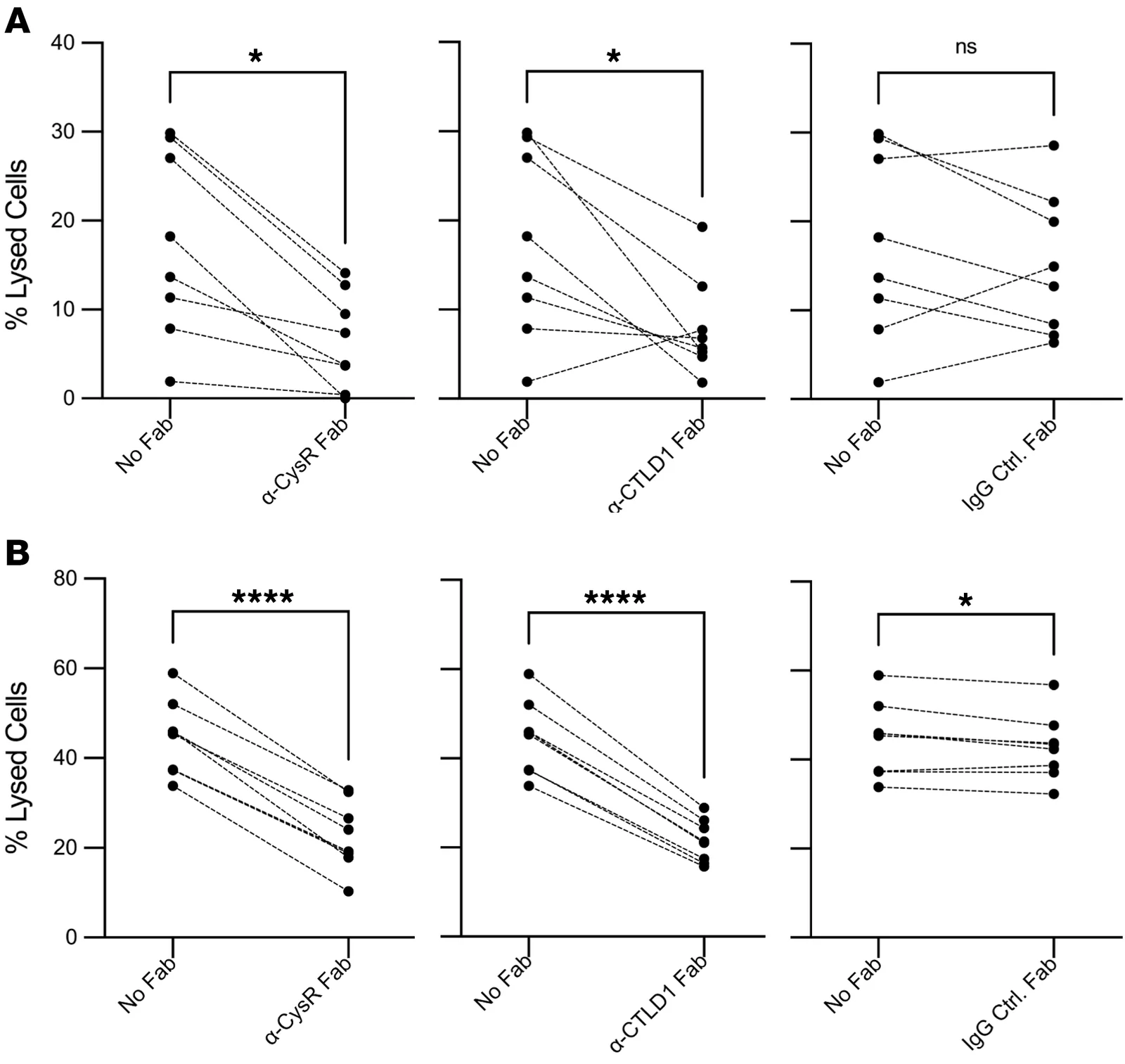
Anti-CysR and anti-CTLD1 Fab fragments reduce patient IgG–induced CDC. (**A**) CDC assays using patient IgG (from patients 1, 2, 4, 6, 7, 8, 9, and 10) with and without preincubation with anti-CysR (left), anti-CTLD1 (middle) Fab fragments, or a control Fab fragment (right) (all Fab fragments were 132 nM). Target cells were PLA2R-AB8/13 podocytes. (**B**) CDC assays using patient IgG (from patients 1, 2, 4, 6, 7, 8, 9, and 10) with and without preincubation with anti-CysR (left), anti-CTLD1 (middle) Fab fragments, or a control Fab fragment (right) (all Fab fragments 132 nM). Target cells were PLA2R-LY cells. *P < 0.05, **P < 0.01, and ****P < 0.0001, by 2-tailed, paired Student’s *t* test.

**Table 2:**
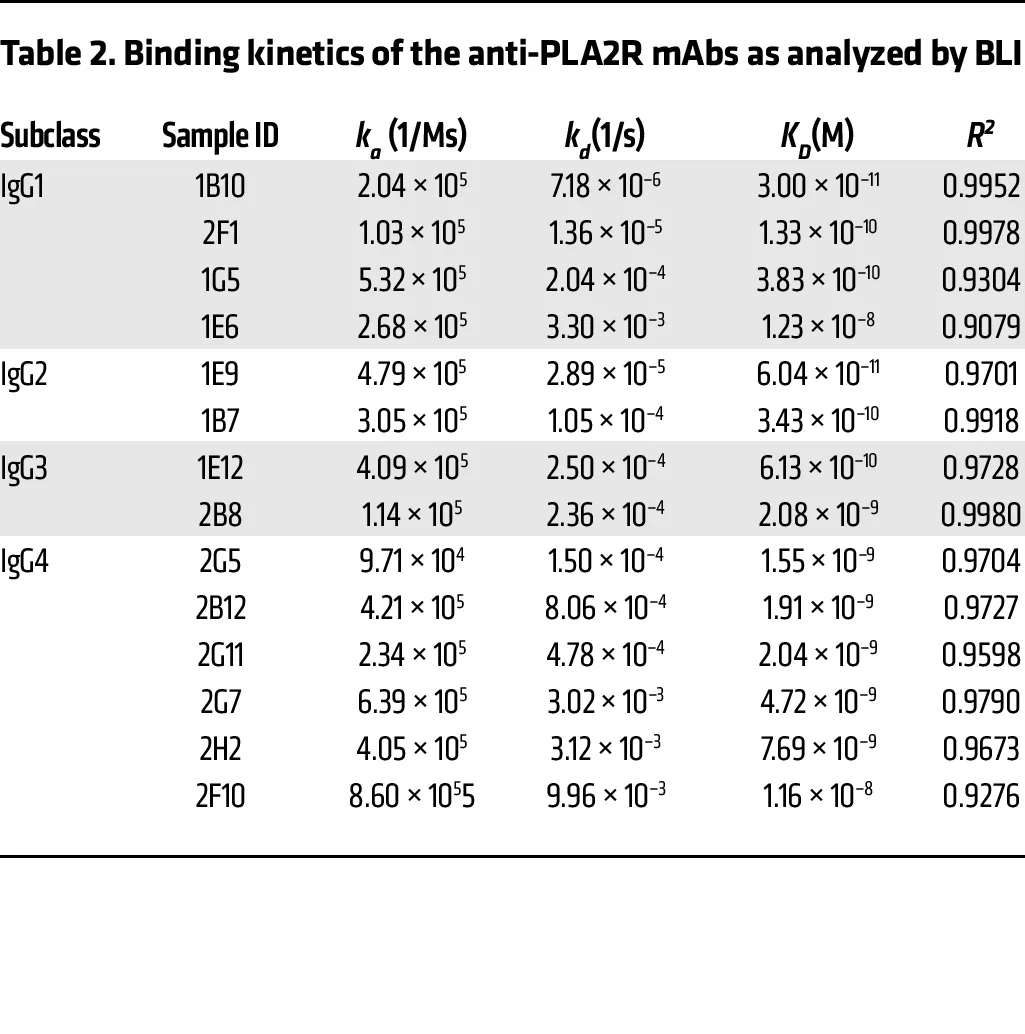
Binding kinetics of the anti-PLA2R mAbs as analyzed by BLI

**Table 1:**
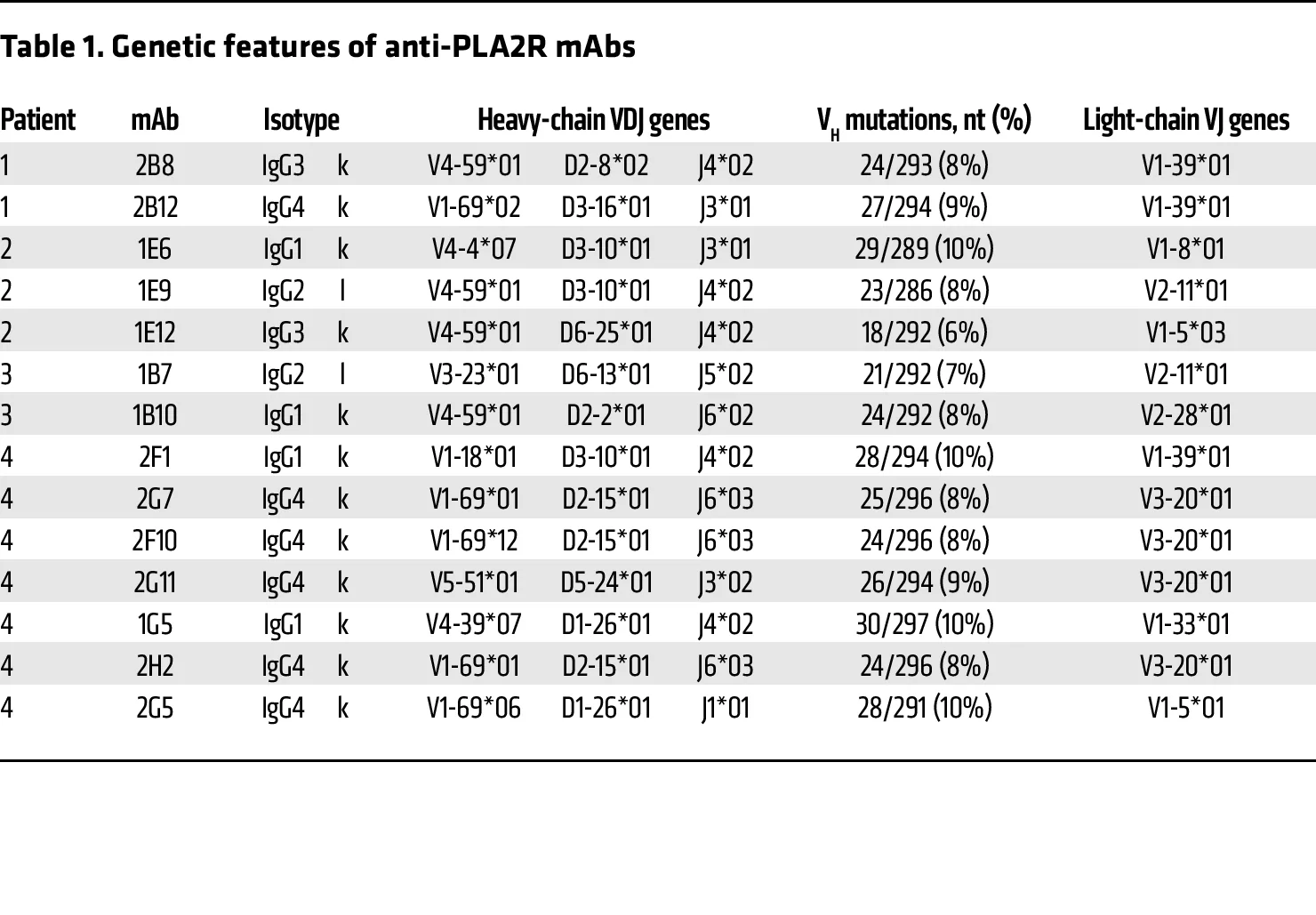
Genetic features of anti-PLA2R mAbs

## Version 1 09/08/2026

In-Press Preview

## Version 2 11/02/2026

Electronic publication
