# Supplementary material for: Anti-PLA2R autoantibodies induce complement activation via IgG subclass–dependent epitope pairings in membranous nephropathy
Source: J Clin Invest. 2026 Sep 8;136(21):e191995. doi: 10.1172/JCI191995 (PMC13722390; doi:10.1172/JCI191995)
Supplement: Supplementary file 1 [file jci-136-191995-s094.pdf]

Supplemental Methods

Generation and production of human IgG antibodies

Human anti-PLA2R monoclonal antibodies were generated using human IgG expression vectors. Antibodies were produced using the F293 transient expression system. Cells were transfected with the corresponding antibody expression plasmids using polyethyleneimine (PEI) at a PEI-to-plasmid ratio of 3:1. Three days after transfection, culture supernatants were collected by centrifugation at 2,000 rpm for 10 min and filtered through a 0.22-μm filter. For antibody purification, Protein G Resin (GenScript) was added to the filtered culture supernatants and incubated overnight at 4°C with rotation. The resin was subsequently collected by centrifugation and washed three times with wash buffer. Bound antibodies were eluted and then dialyzed against 20 mM Tris-HCl, with the dialysis buffer replaced every 18 h for a total of three exchanges. Purified antibodies were finally filtered through a 0.22-μm filter and stored at −80°C until use.

Generation of mouse IgG2a versions

To enable passive transfer experiments in mice, chimeric antibodies comprising the human variable regions and a mouse IgG2a constant region were generated. The heavy-chain variable domains of the selected anti-PLA2R clones were recloned in frame with a mouse IgG2a heavy-chain constant region, and the corresponding light chains were paired with a mouse kappa constant region, in the pcDNA3.4 expression vector. Antibodies were produced by GenScript (a contract research organization) using their standard expression and purification workflow. In brief, constructs were expressed by transient transfection of CHO cells and antibodies were purified from culture supernatants by Protein A affinity chromatography followed by preparative size-exclusion chromatography, yielding a purity greater than 95% as determined by SDS-PAGE and HPLC-SEC. Purified antibodies were buffer-exchanged into PBS and sterile-filtered (0.22 μm) prior to in vivo use.

Expression of full-length and truncated PLA2R

Freestyle 293 cells were transfected with pSectag2B-sPLA2R and cultured in serum-free Freestyle 293 medium for 3 days. The medium was then concentrated and applied to nickel-bead (Sigma Aldrich) for purification using affinity chromatography. After binding to the beads, elution was carried out using imidazole, followed by dialysis in 20 mM Tris for 3 days. The purified sPLA2R was analyzed using SDS-PAGE and western blotting with anti-myc tag antibodies (Santa Cruz Biotechnology, 9E10, sc-40) and anti-PLA2R antibodies (Sigma Aldrich, polyclonal, HPA012657) (Fig. S1B).

Immunofluorescence of podocytes and HEK293 cells

HEK293 and differentiated podocytes grown on 20 mm round coverslips were washed three times with PBS and fixed in 4% formaldehyde for 15 minutes. After three more washes with PBS, cells were blocked with 3% BSA for 1 hour, followed by two washes with PBS. The primary antibody (anti-PLA2R mAb, 1 μg/ml) was diluted in 3% BSA solution and added to the cells, which were incubated overnight at 4°C. The

next day, cells were washed three times with PBS, and Alexa 594-conjugated mouse anti-human IgG secondary antibody (Jackson ImmunoResearch, 209-585-082) was added at a dilution of 1:500 for 1 hour at room temperature. Cells were washed three times with PBS, then stained with a 1:2000 dilution of Hoechst 33342 (Invitrogen) and a 1:400 dilution of Phalloidin (Invitrogen) for 5 minutes. Finally, the cells were washed three times with PBS and mounted onto microscope slides. Images were acquired on a Zeiss.

Flow cytometry to identify mAb binding to mPLA2R

To assess the binding ability of monoclonal anti-PLA2R antibodies, 1x106 mPLA2R-podocytes were incubated with anti-PLA2R mAb (1 µg/ml) for 1 hour on ice. Following incubation, APC-conjugated anti-human IgG Fc antibody (BioLegend, M1310G05, 410712) was added, and the cells were incubated for an additional 30 minutes on ice. Afterward, the signal was detected using flow cytometry (BD Verse).

Supplemental Figures

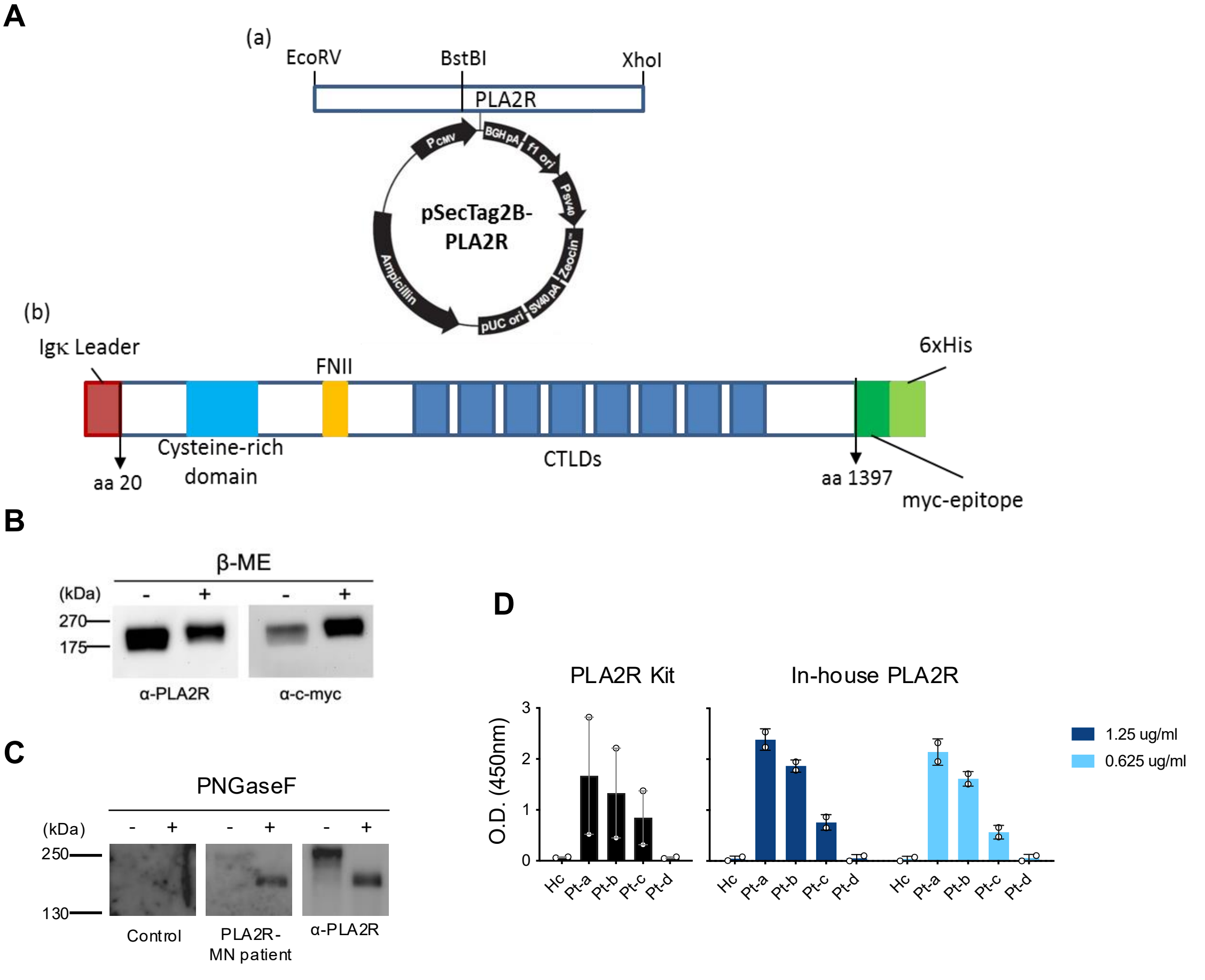

Fig. S1. Cloning and characterization of recombinant soluble PLA2R.

(A) Scheme of the recombinant soluble PLA2R protein with myc and 6xHis tags at the C-terminus.

(B) Western blot showing the reactivity of soluble PLA2R with polyclonal anti-PLA2R antibodies (α-PLA2R) and anti-myc antibodies (α-c-myc) in the presence or the absence of β-mercaptoethanol as a reducing agent.

(C) Western blot showing the reactivity of soluble PLA2R with healthy control plasma (control), PLA2R-MN patient plasma, and polyclonal anti-PLA2R antibodies (α-PLA2R) in the presence or absence of the deglycosylating reagent PNGaseF.

(D) Comparison of binding O.D. values for healthy control plasma (Hc) and PLA2R-MN patient plasma (Pt-a, -b, -c, -d) using a commercial kit (EUROIMMUN) (black bar) versus an in-house anti-PLA2R ELISA assay (reacted PLA2R concentration 1.25 µg/ml (dark blue bars) and 0.625 µg/ml (blue bars)). The data are presented as the mean and S.D. for two independent experiments.

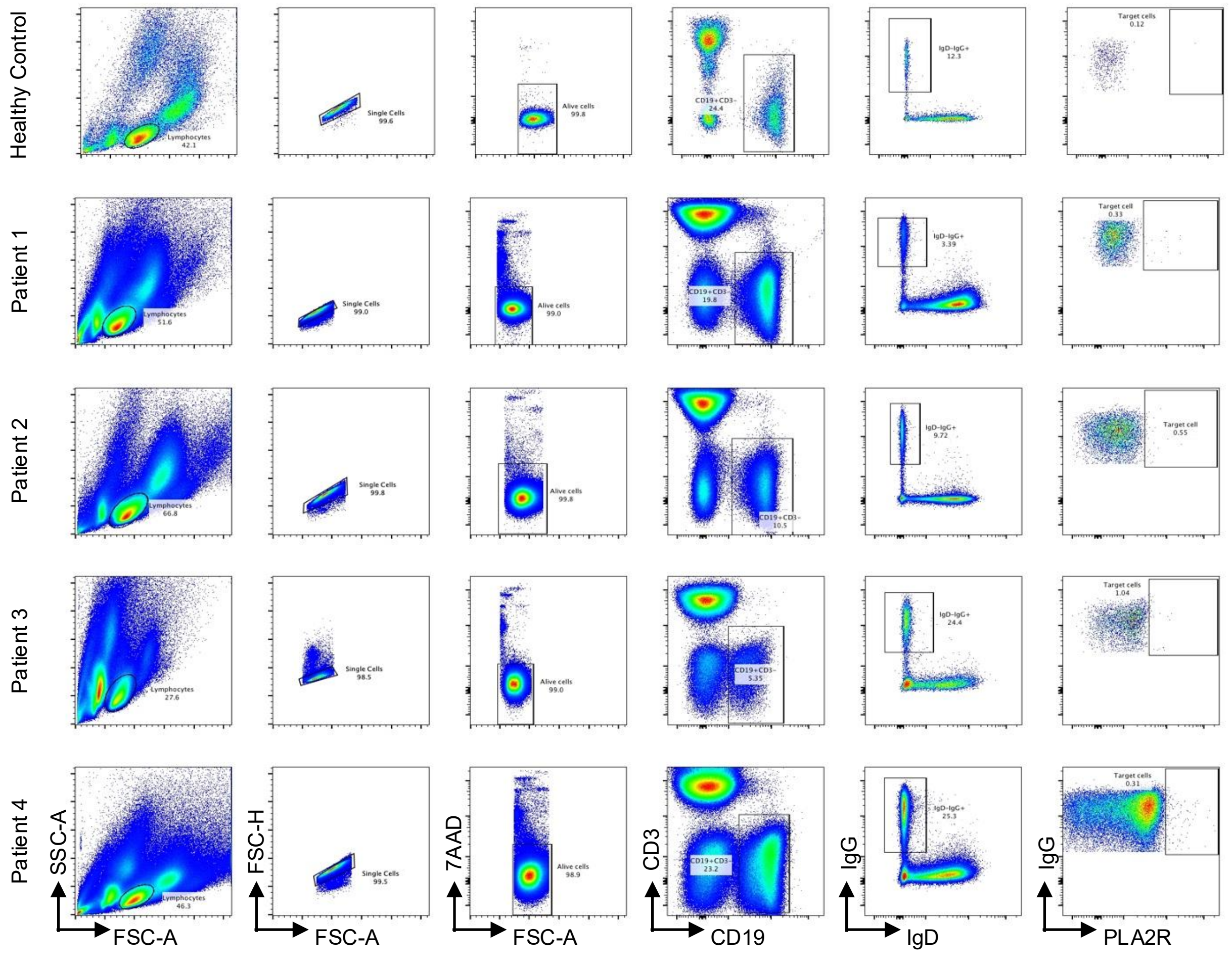

Fig. S2. Flow cytometry panel of anti-PLA2R-positive B cells in peripheral blood mononuclear cells (PBMCs) from 4 MN Patients.

Isolation of individual PLA2R-specific IgG^{+}CD19^{+} B cells from patients with anti-PLA2R positive membranous nephropathy. The flow cytometry plots illustrate the gating strategy employed to identify human PLA2R-specific B cells PBMCs. The outlined black boxes represent each step in the gating process. Lymphocytes, distinguished based on forward scatter (FSC) and side scatter (SSC) characteristics, were further scrutinized using FCS-A versus FSC-H and stained with 7-AAD to exclude doublets and dead cells. Subsequently, CD19^{+}CD3^{-} cells were selected, followed by IgG^{+}IgD^{-} cells, to enrich the population of B cells specifically binding to PLA2R, which were labeled with an APC-conjugated antibody. Finally, PLA2R^{+} cells were sorted from the IgG^{+} subsets.

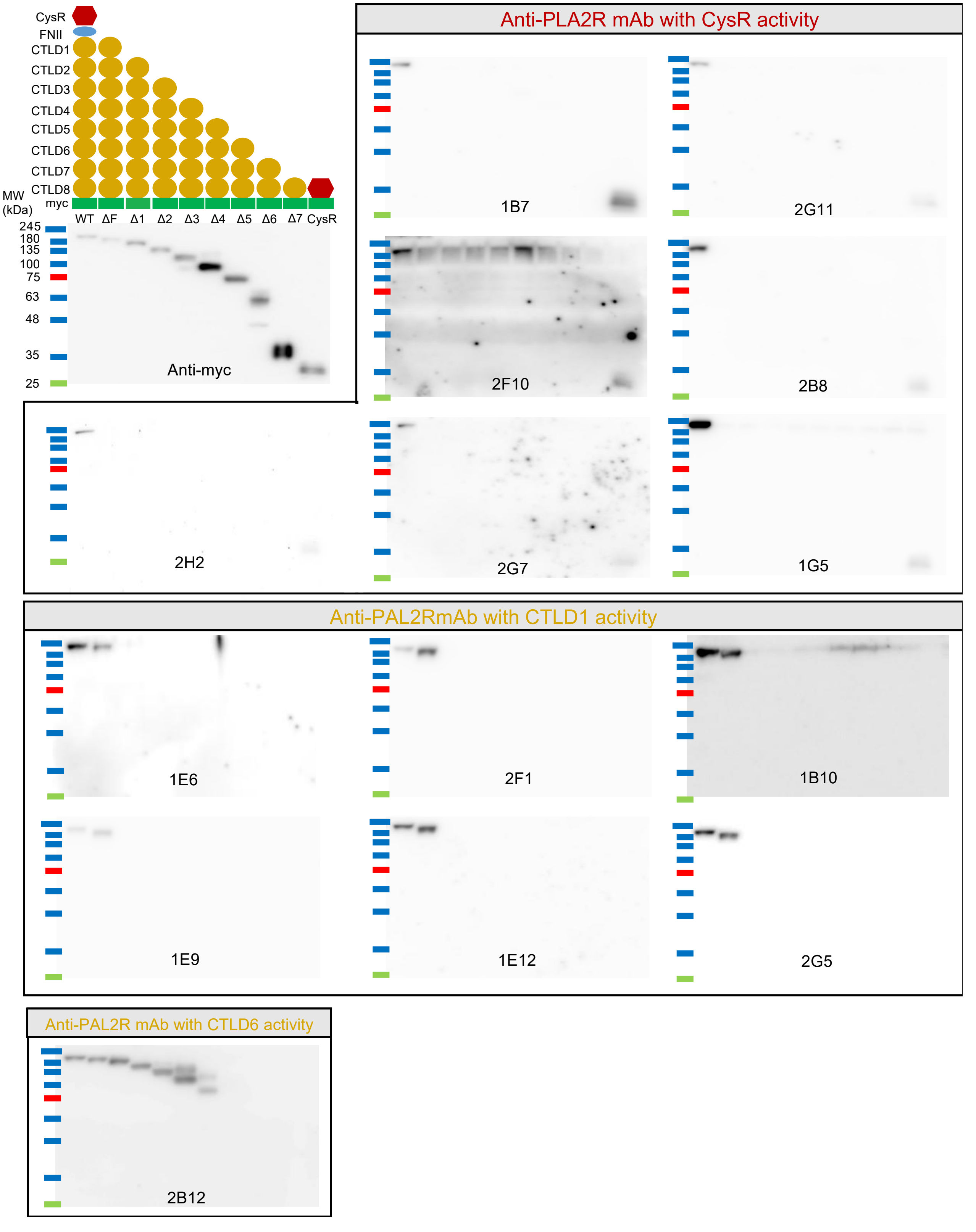

Fig. S3. Binding domain characterization of all anti-PLA2R monoclonal antibodies.
The anti-myc, 1B10, 2B8, and 2B12 blots shown here were previously presented in Figure 2A. Scheme of the PLA2R structure and the PLA2R mutants used to identify the epitope regions targeted by the patient-derived mAbs (upper panel). Representative Western blot analyses of recombinant PLA2R and PLA2R mutants using anti-PLA2R mAbs (lower panel). The results for three mAbs targeting different binding domains (CysR, CTLD1, and CTLD6) are highlighted by black boxes.

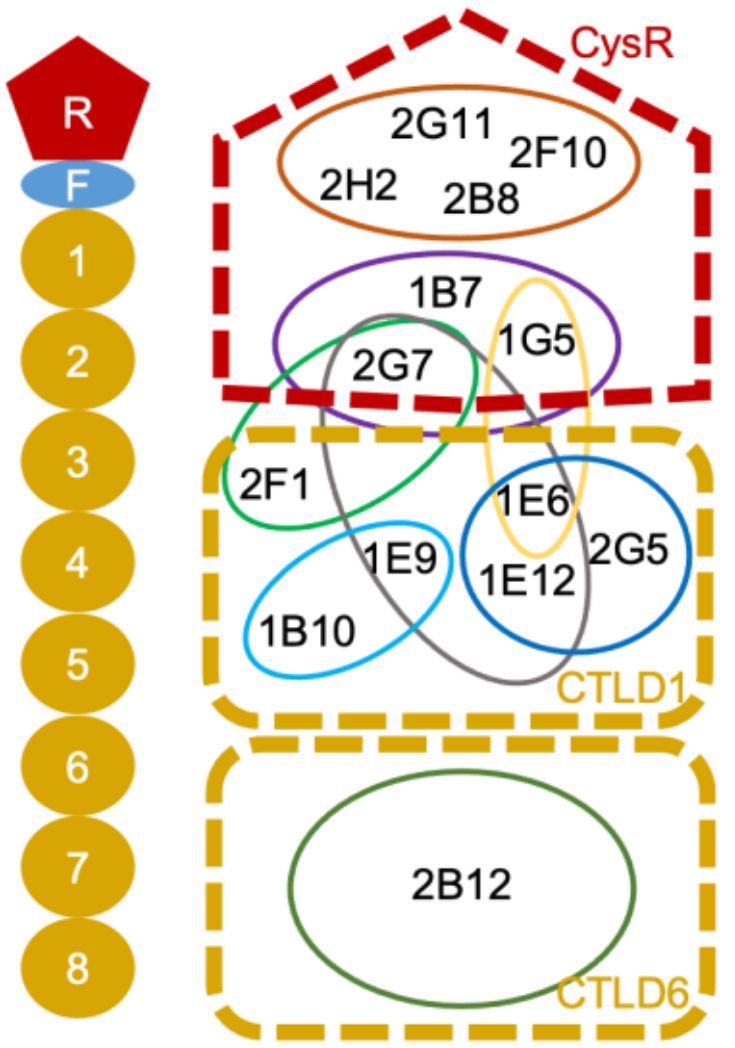

Fig. S4. Summary of the binding characteristics of anti-PLA2R mAbs based on Western blotting and epitope binning by BLI.

Scheme of mAbs binding domain. Dashed boxes mean mAbs binding domain which definition by Western blot with sPLA2R mutants. mAb circled together mean mAbs exhibiting co-binding PLA2R in the BLI assays with sPLA2R.

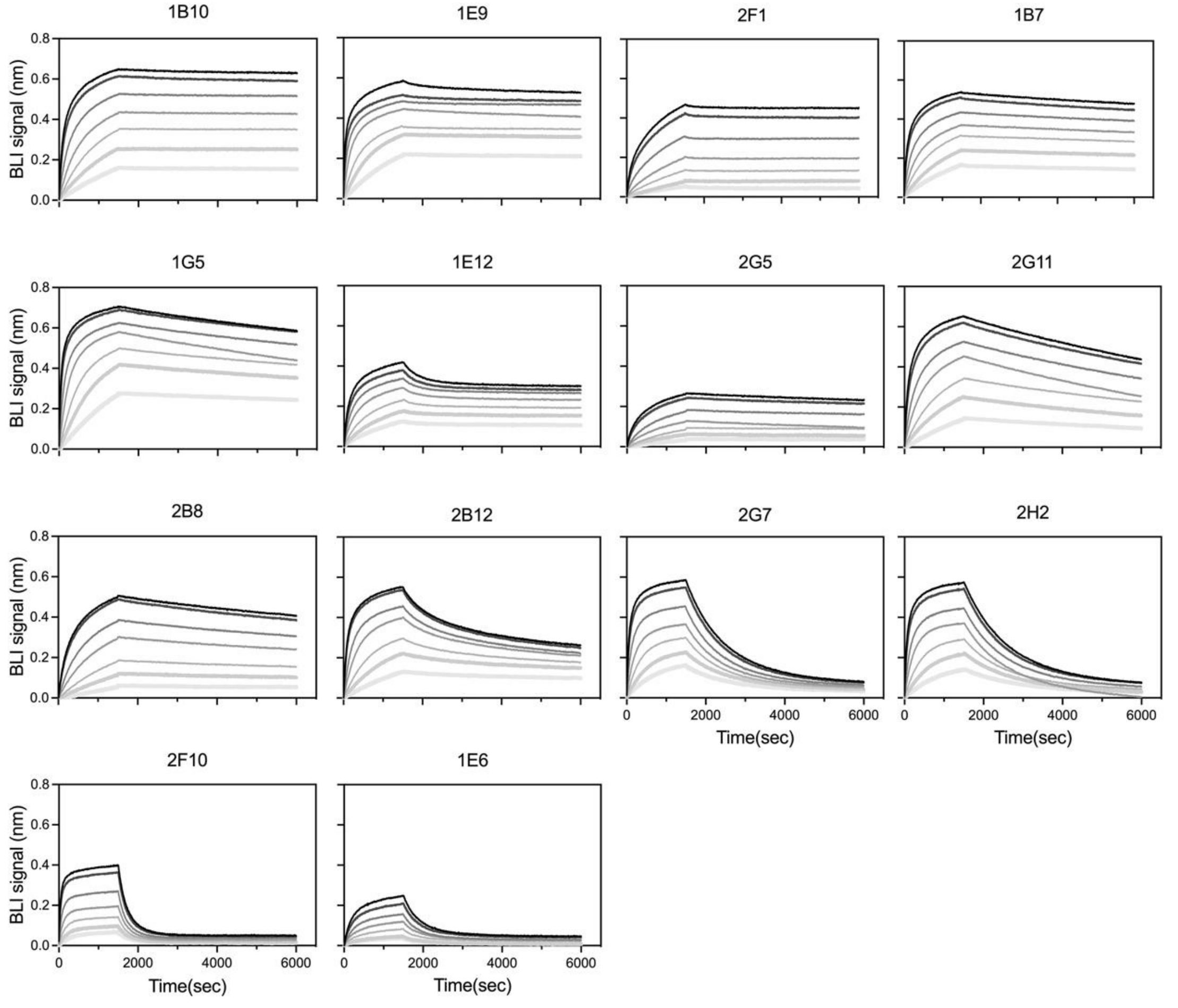

Fig. S5. Individual binding kinetics of the anti-PLA2R mAbs to soluble PLA2R.

Sensorgrams showing the binding of 14 monoclonal anti-PLA2R antibodies (750 ng/ml) to various concentrations of recombinant soluble PLA2R protein, specifically 0, 3.125, 6.25, 12.5, 25, 50, 100, and 125 nM, as analyzed using BLI. The resulting association and dissociation curves were processed by subtracting the data from a blank control (running the buffer without PLA2R) to correct for background signals.

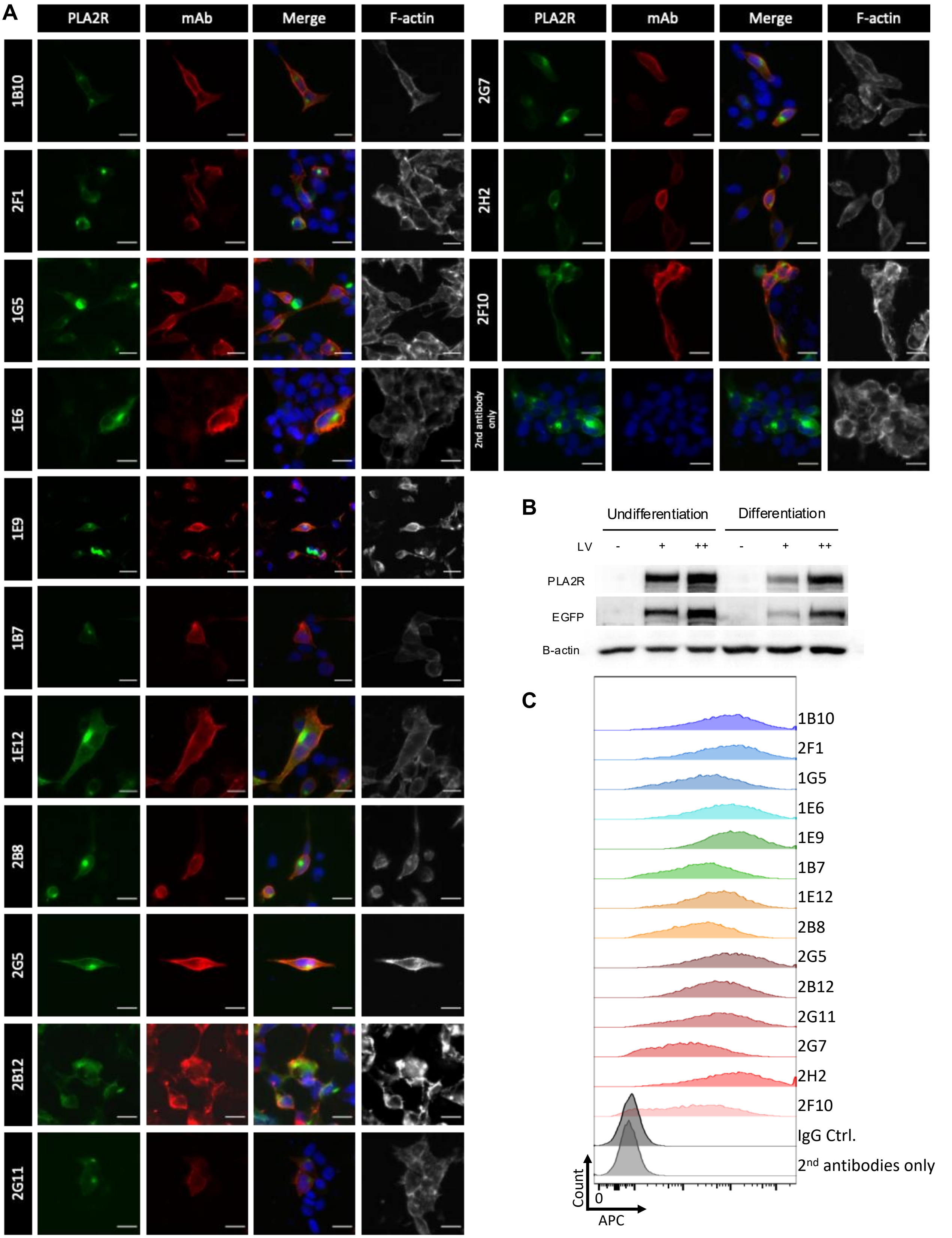

Fig. S6. Binding of anti-PLA2R mAbs to membrane PLA2R on cell surfaces.

(A) Representative immunofluorescence images of the anti-PLA2R mAbs (red) reactive with membrane PLA2R (green) on overexpressing mPLA2R-EGFP HEK293 cells. Hoechst staining (blue) indicates nuclei, and phalloidin (gray) highlights F-actin structures. Scale bar, 20 µm.

(B) Western blot assays showing podocyte cell line (AB8/13) was transfected by a lentivirus (LV) to overexpress mPLA2R-EGFP fusion proteins were present in both differentiated and non-differentiated podocytes.

(C) Flow cytometry analysis showing the binding of anti-PLA2R mAbs to mPLA2R-expressing podocytes, detected using APC-conjugated anti-human IgG Fc F(ab')$_2$ fragments to identify mAbs.

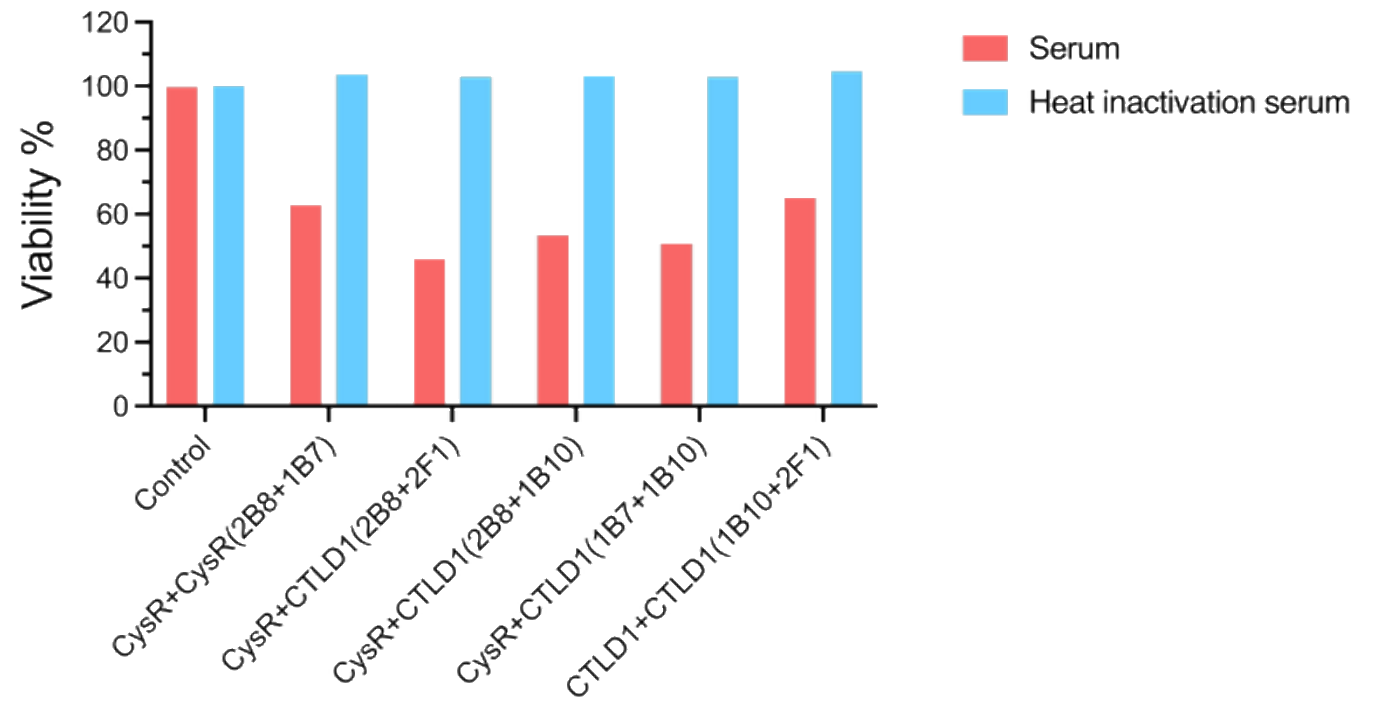

Fig. S7. CDC assays using heat-inactivated serum as a source of complement.
CDC assays using a CysR-reactive mAb (2B8 or 1B7 mAb, 1 μg/ml) paired with a CTLD1-reactive mAb (1B10 or 2F1 mAb) or two CysR-reactive mAbs or two CTLD1-reactive mAbs.

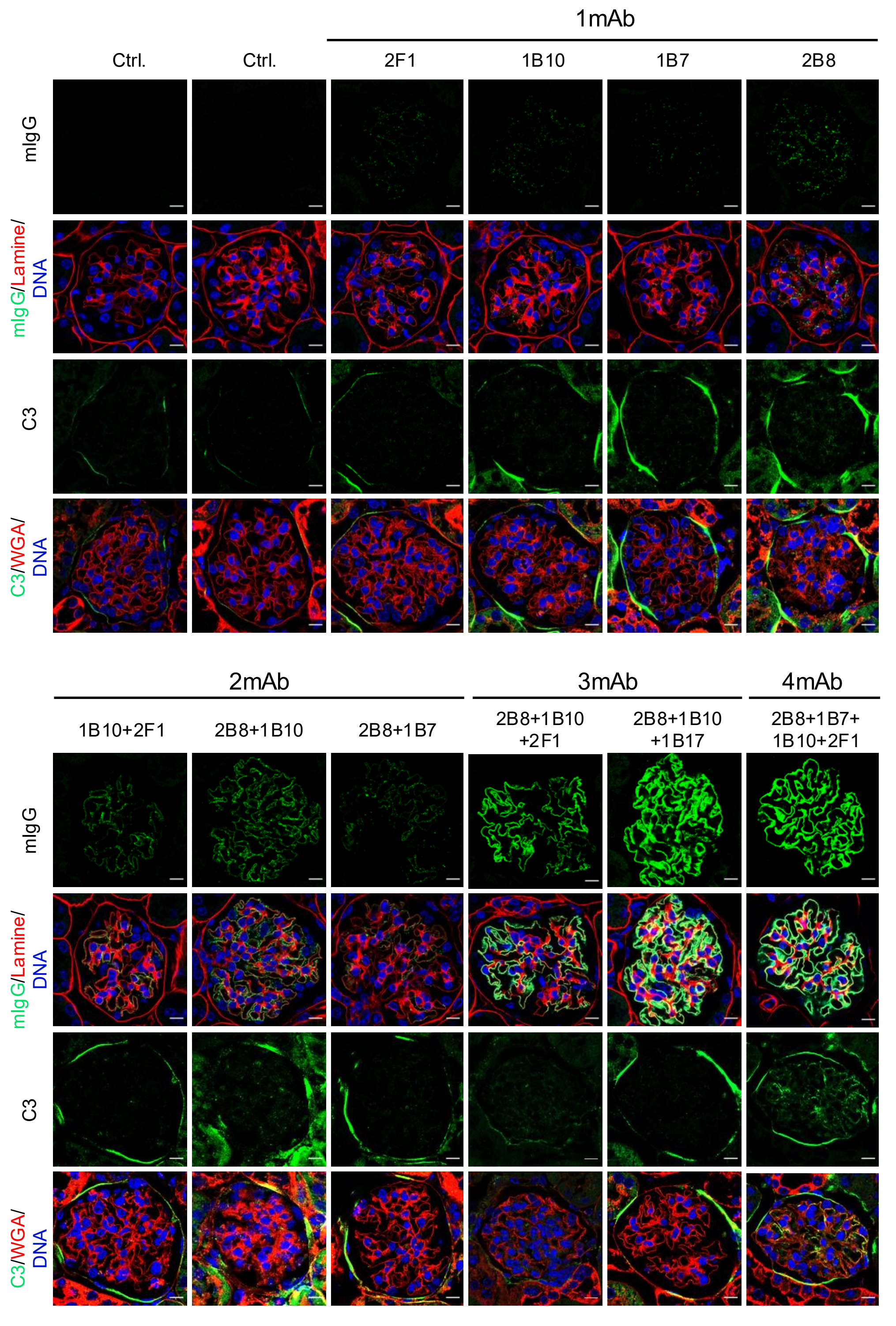

Fig. S8. Anti-PLA2R mAb transfer to hPLA2R-transgenic mice.
Representative immunofluorescence images of glomeruli from hPLA2R1-transgenic Rag2–/– mice mice 10 days after antibody transfer showing mouse IgG (mIgG) co-stained with the GBM marker laminin, and C3 co-stained with wheat germ agglutinin (WGA). Mice were injected with control IgG (n = 4) or anti-PLA2R mAbs (1B10, 2B8, 1B7, 2F1) administered as different combinations comprising 1 mAb (n = 12), 2 mAbs (n = 9), 3 mAbs (n = 6), or 4 mAbs (n = 3). Scale bars, 10 µm.

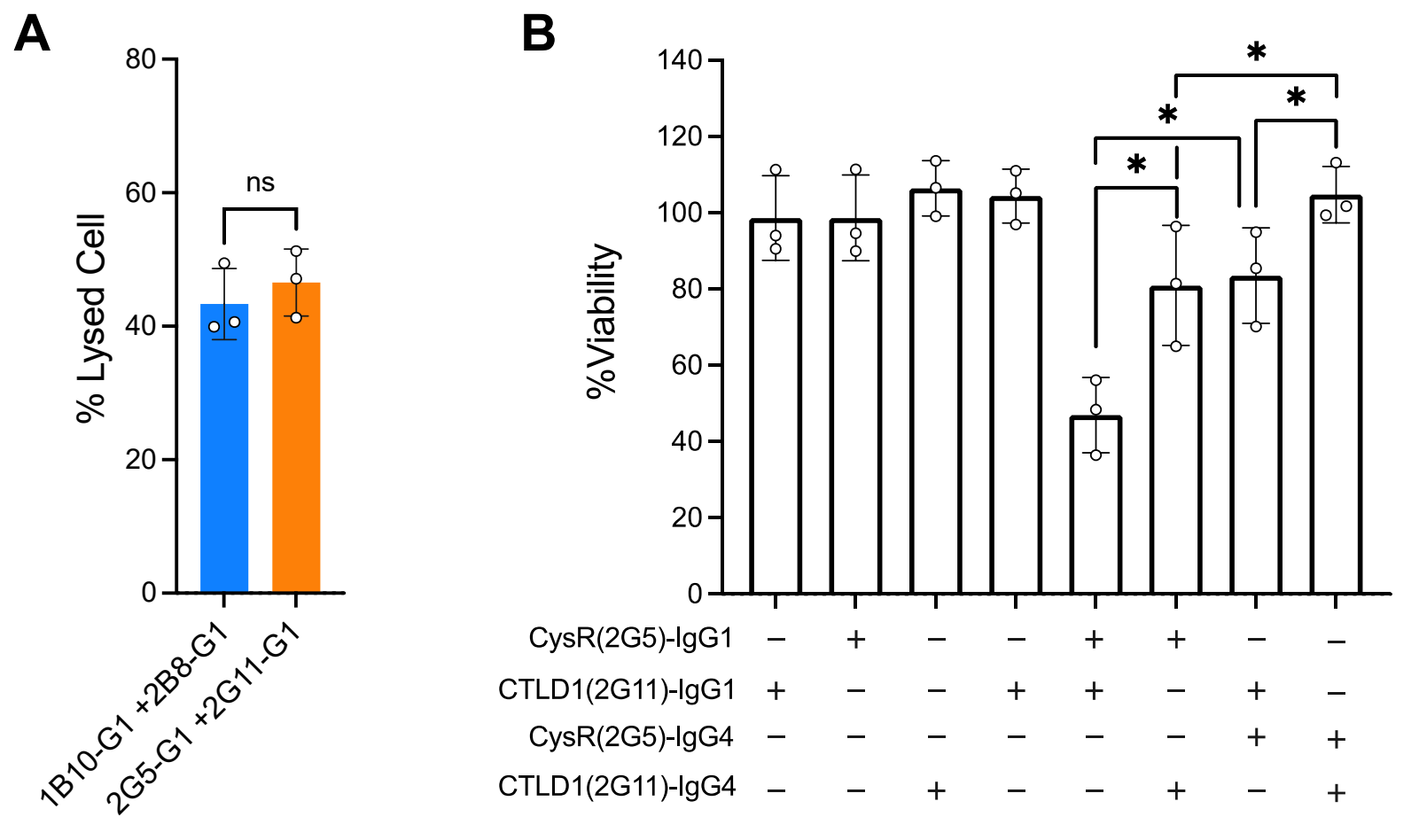

Fig. S9. Fc replacement of the IgG4 mAbs with IgG1 induced the epitope pairing-mediated CDC synergy.

(A) CDC assays using the the CTLD1-reactive mAb (1B10 or 2G5 mAb, 1 μg/ml) paired with CysR-reactive mAb (2B8 or 2G11 mAb, 1 μg/ml) on IgG1 backbone.

(B) CDC assays using the the the CTLD1-reactive mAb (2G5 mAb, 1 μg/ml) and CysR-reactive mAb (2G11 mAb, 1 μg/ml) on IgG1 and IgG4 Fc backbones.

Data are shown as Mean ± SD, n = 3. Statistical analysis were performed using one-way ANOVA followed by Dunnett's multiple-comparisons test. ns, not significant. *$P < 0.05$.

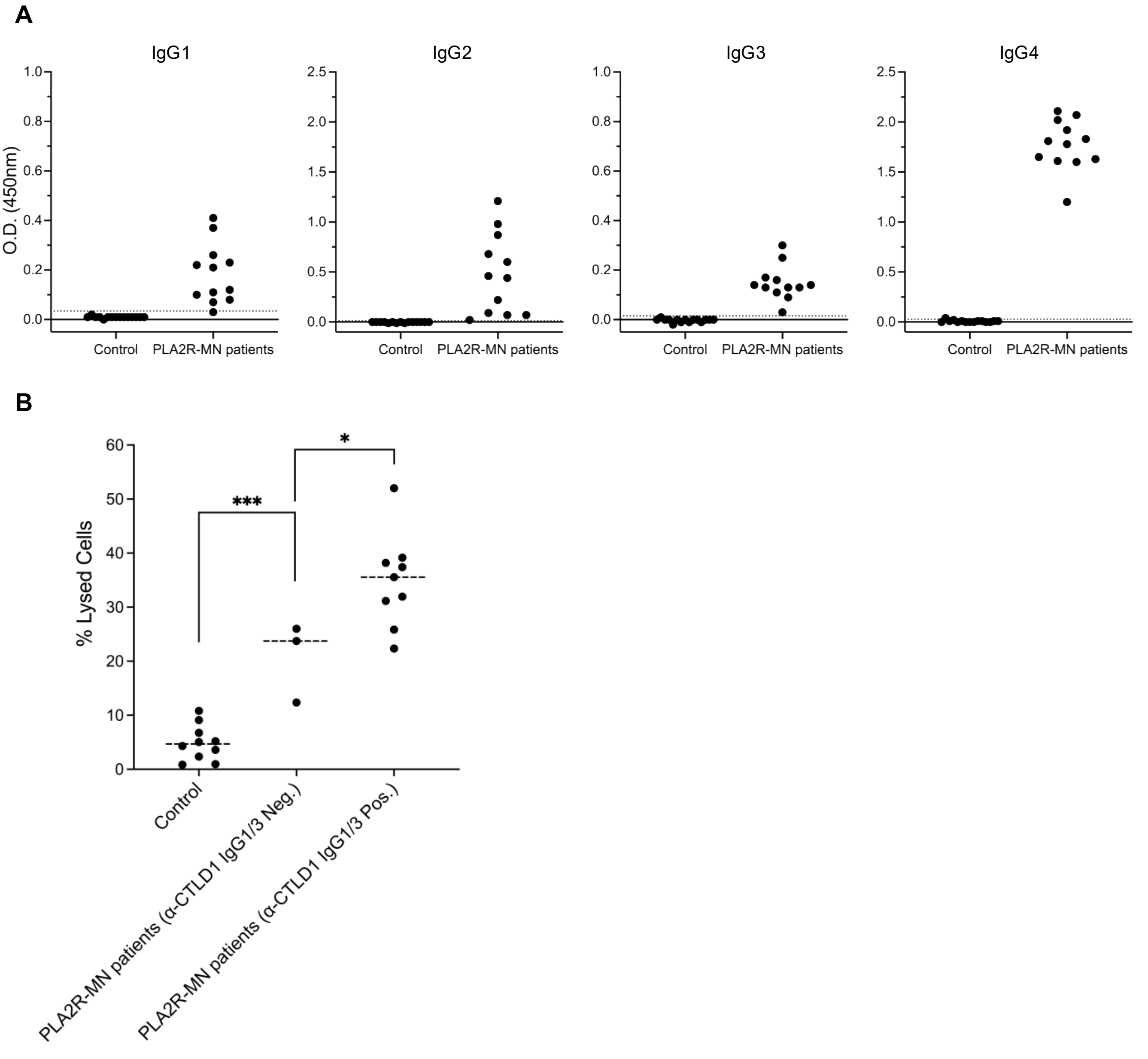

Fig. S10. Distribution of MN patient IgGs subclass and group MN patient by anti-PLA2R epitope distribution.

(A) ELISA showing the IgG subclasses of individual anti-PLA2R antibodies present in the plasma of 12 PLA2R-MN patients. The negative threshold (dotted line) represents the mean plus 3 SD of plasma samples from healthy controls (control).

(B) CDC assays using the total IgG fraction from healthy individuals (control) and patients with PLA2R-associated pMN on PLA2R-expressing podocytes, separated into two groups; Undetectable anti-CTLD1 IgG1/3 (PLA2R-MN patients (α-CTLD1 IgG1/3 Neg.)) (Pt. 5, 12, 13) and detectable anti-CTLD1 IgG1/3 (PLA2R-MN patients (α-CTLD1 IgG1/3 Pos.)) (Pt. 1, 2, 4, 6, 7, 8, 9, 10, 11)). Data are presented as the mean. Statistical analyses were performed using a two-tailed unpaired Student's t-test. *$P < 0.05$ ***$P < 0.001$.
