# Supplementary material for: Anti-PLA2R autoantibodies induce complement activation via IgG subclass–dependent epitope pairings in membranous nephropathy
Source: J Clin Invest. 2026 Sep 8;136(21):e191995. doi: 10.1172/JCI191995 (PMC13722390; doi:10.1172/JCI191995)
Supplement: Supplementary file 2 [file jci-136-191995-s095.pdf]

Fig. 1. D part1

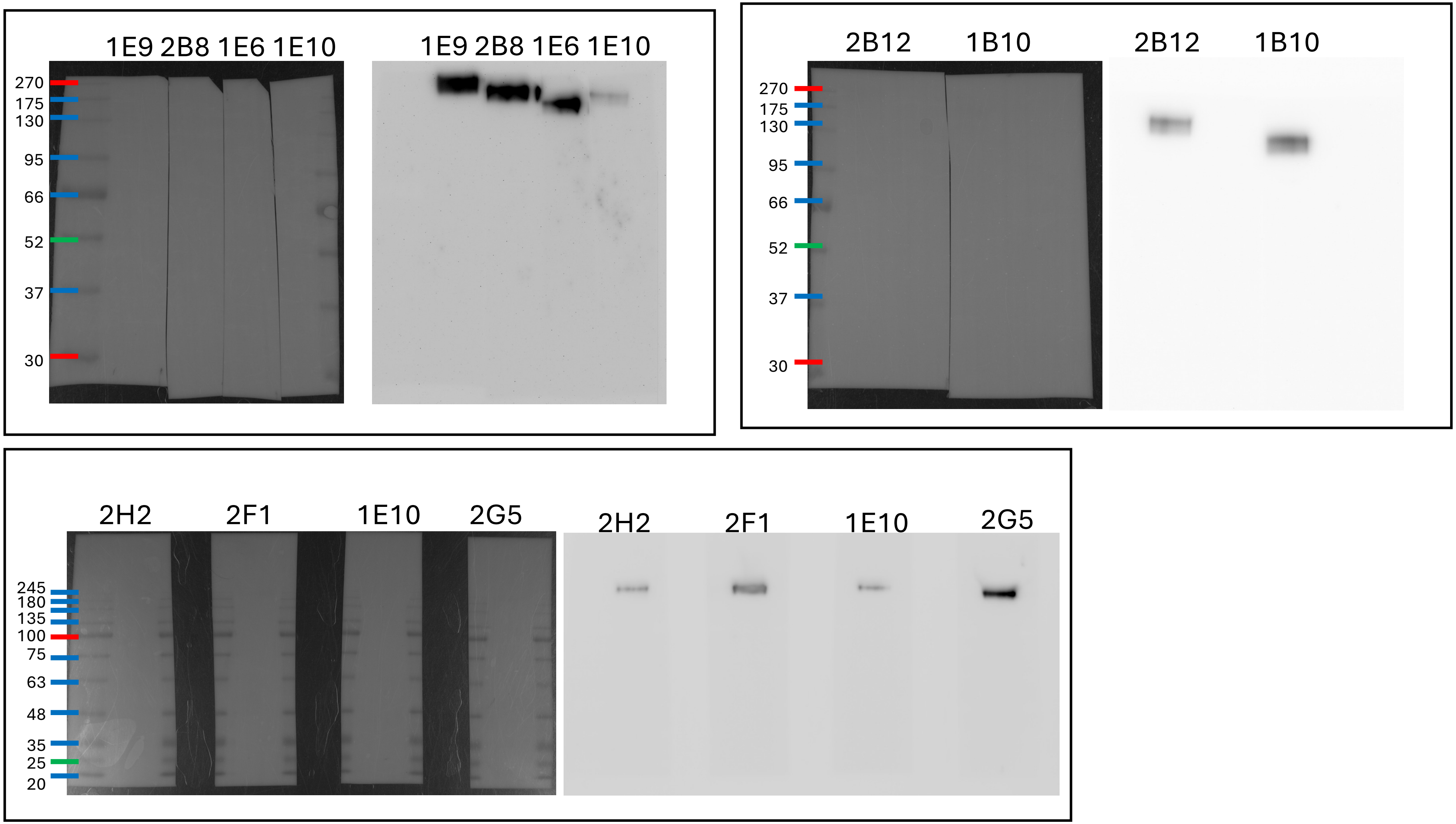
1E9 2B8 1E6 1E10
270
175
130
95
66
52
37
30
1E9 2B8 1E6 1E10
2B12 1B10
270
175
130
95
66
52
37
30
2B12 1B10
2H2 2F1 1E10 2G5
245
180
135
100
75
63
48
35
25
20
2H2 2F1 1E10 2G5

Fig. 1. D part2

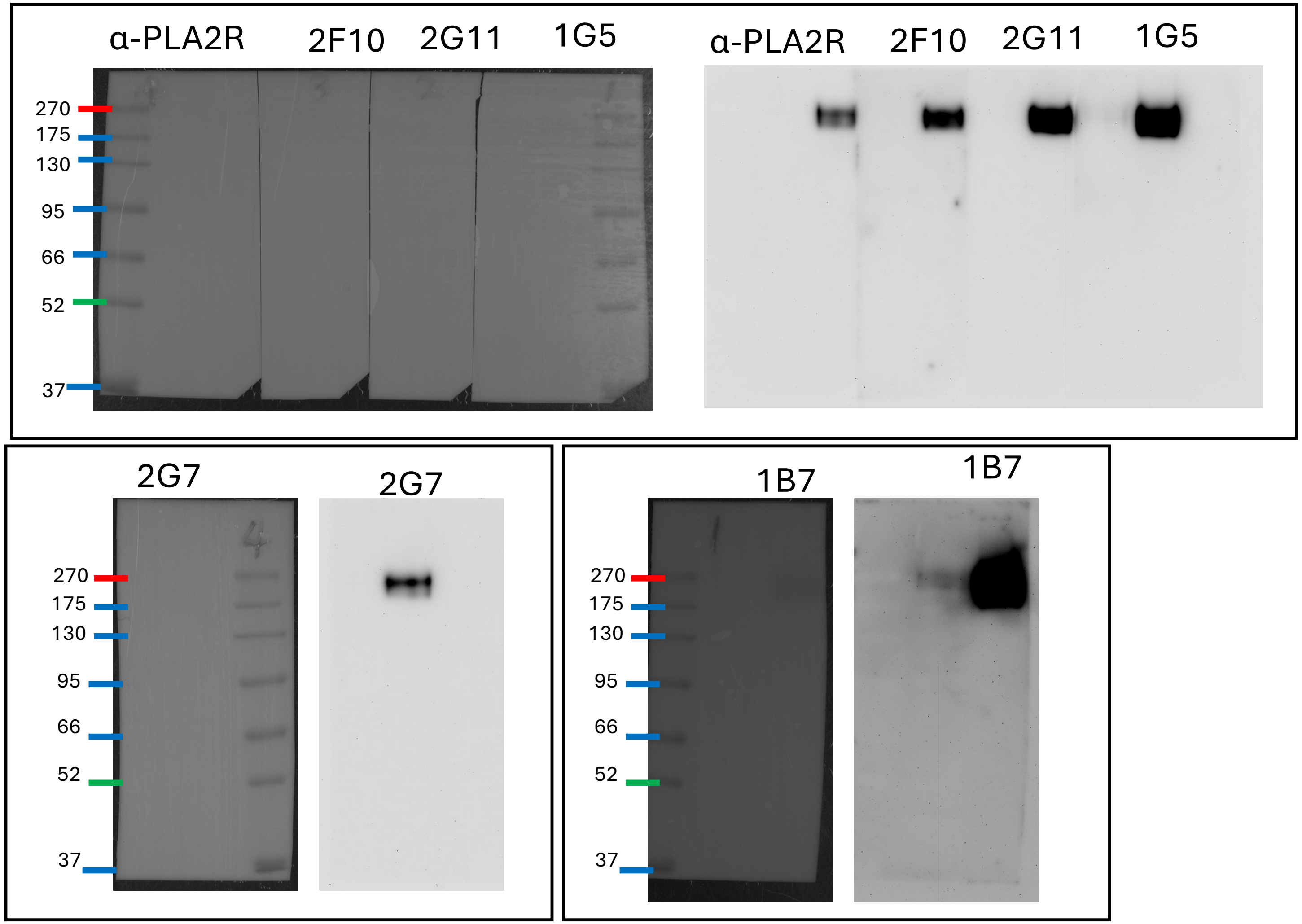
α-PLA2R
2F10
2G11
1G5
270
175
130
95
66
52
37
α-PLA2R
2F10
2G11
1G5
2G7
2G7
270
175
130
95
66
52
37
1B7
1B7
270
175
130
95
66
52
37

Fig. S1. B

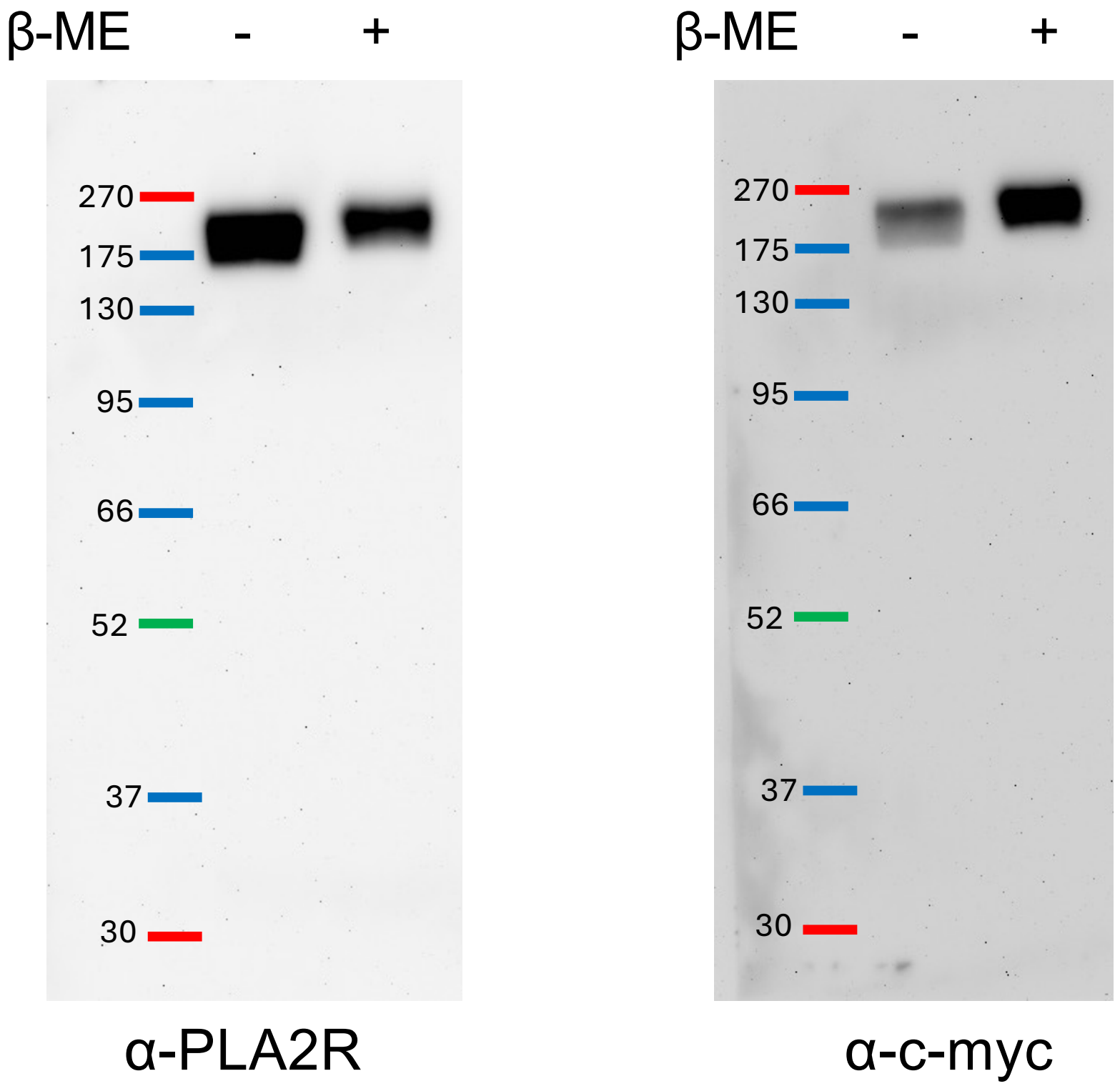
β-ME - +
270
175
130
95
66
52
37
30
α-PLA2R
β-ME - +
270
175
130
95
66
52
37
30
α-c-myc

Fig. S1. C

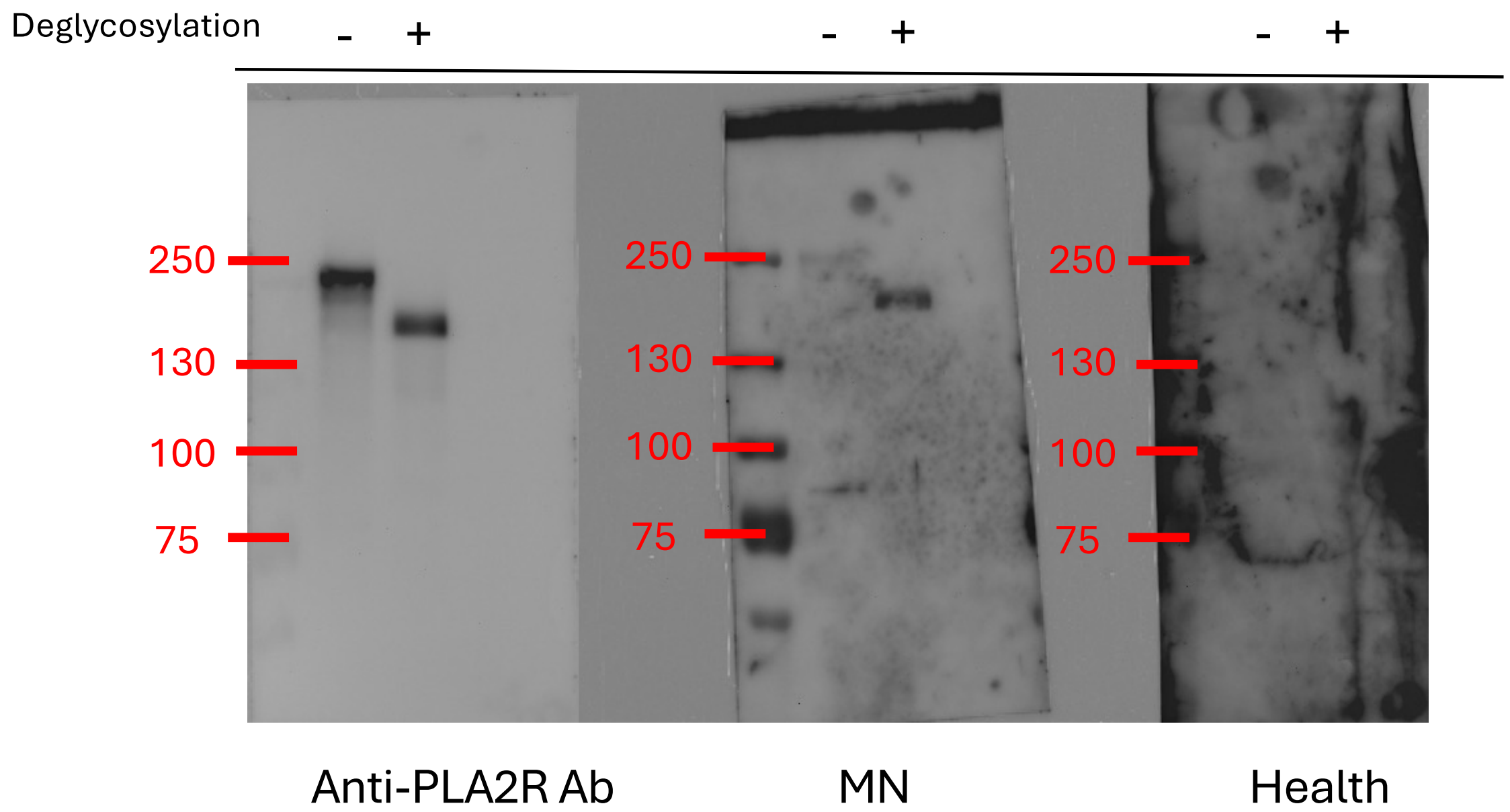
Deglycosylation
-
+
-
+
-
+
250
130
100
75
250
130
100
75
250
130
100
75
Anti-PLA2R Ab
MN
Health

Fig. S6. B

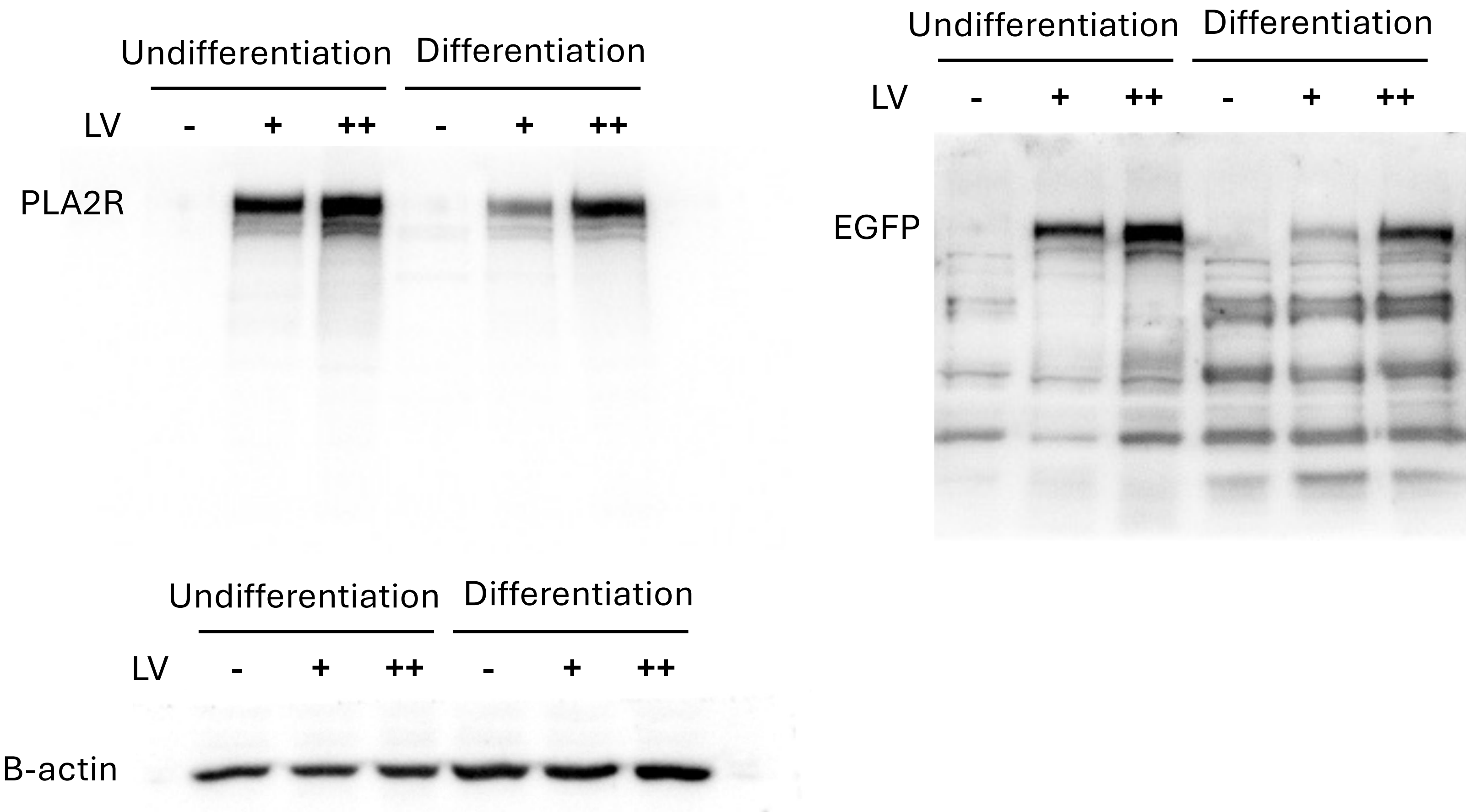
Undifferentiation
Differentiation
LV - + ++ - + ++
PLA2R
Undifferentiation
Differentiation
LV - + ++ - + ++
EGFP
Undifferentiation
Differentiation
LV - + ++ - + ++
B-actin
